# Dragon fruit detection in natural orchard environment by integrating lightweight network and attention mechanism

**DOI:** 10.3389/fpls.2022.1040923

**Published:** 2022-10-20

**Authors:** Bin Zhang, Rongrong Wang, Huiming Zhang, Chenghai Yin, Yuyang Xia, Meng Fu, Wei Fu

**Affiliations:** ^1^ School of Information and Communication Engineering, Hainan University, Haikou, China; ^2^ Mechanical and Electrical Engineering College, Hainan University, Haikou, China; ^3^ College of Mechanical and Electrical Engineering, Shihezi University, Shihezi, China

**Keywords:** dragon fruit, object detection, YOLOv5s, lightweight network, mechanism of attention

## Abstract

An improved lightweight network (Improved YOLOv5s) was proposed based on YOLOv5s in this study to realise all-weather detection of dragon fruit in a complex orchard environment. A ghost module was introduced in the original YOLOv5s to realise the lightweight of the model. The coordinate attention mechanism was joined to make the model accurately locate and identify the dense dragon fruits. A bidirectional feature pyramid network was built to improve the detection effect of dragon fruit at different scales. SIoU loss function was adopted to improve the convergence speed during model training. The improved YOLOv5s model was used to detect a dragon fruit dataset collected in the natural environment. Results showed that the mean average precision (*mAP*), precision (*P*) and recall (*R*) of the model was 97.4%, 96.4% and 95.2%, respectively. The model size, parameters (Params) and floating-point operations (FLOPs) were 11.5 MB, 5.2 M and 11.4 G, respectively. Compared with the original YOLOv5s network, the model size, Params and FLOPs of the improved model was reduced by 20.6%, 18.75% and 27.8%, respectively. Meanwhile, the *mAP* of the improved model was improved by 1.1%. The results prove that the improved model had a more lightweight structure and better detection performance. Moreover, the average precision (*AP*) of the improved YOLOv5s for dragon fruit under the front light, back light, side light, cloudy day and night was 99.5%, 97.3%, 98.5%, 95.5% and 96.1%, respectively. The detection performance met the requirements of all-weather detection of dragon fruit and the improved model had good robustness. This study provides a theoretical basis and technical support for fruit monitoring based on unmanned aerial vehicle technology and intelligent picking based on picking robot technology.

## 1 Introduction

People love Dragon fruit because of its high nutritional value, constipation prevention, detoxification, blood glucose reduction, antioxidants and other effects (Attar et al., 2022). The field management of this tropical fruit is labour-intensive. Thus, studying disease and pest monitoring for the fruit based on unmanned aerial vehicle (UAV) technology and intelligent picking based on picking robot technology is very important. Fruit and vegetable object detection in the natural orchard environment is a key technology for monitoring and picking fruit pests and diseases (Tang et al., 2020; Zheng et al., 2021). Given the complex environmental information, such as uneven light intensity and overlapping occlusion between branches and leaves and fruits in dragon fruit orchards (Jiang et al., 2012; Chu and Chang, 2020), studying a method that can accurately detect dragon fruit in complex environments for efficient and automatic all-weather fruit monitoring and picking of dragon fruit is of great research value and practical significance.

Researchers at home and abroad have recently achieved certain results in the field of fruit and vegetable object detection, and a variety of object detection methods proposed have been applied to fruit and vegetable detection tasks in natural scenarios (Behera et al., 2018; Jiang et al., 2019; He et al., 2020; Yu et al., 2021; Jiang et al., 2022). These methods are mainly based on traditional image processing methods and deep learning algorithms (Saleem et al., 2021). Traditional image processing methods are mainly based on the colour, shape and texture of fruits and vegetables, which have been widely used to recognise citrus (Kurtulmus et al., 2011; Lu et al., 2018), apple (Rakun et al., 2011; Linker et al., 2012; Sun et al., 2019), pineapple (Chaivivatrakul and Dailey, 2014) and mango (Payne et al., 2013). However, these methods have high environmental requirements. When the orchard light is uneven and occlusions are found between fruits, recognition accuracy is significantly reduced. With the rapid development of deep learning, a convolutional neural network (CNN) algorithm has been applied to fruit and vegetable object detection, achieving good results. Typical studies are as follows: Sun et al. (2018) proposed an improved Faster-RCNN for tomato recognition, which adopted ResNet50 as the feature extraction network and used the k-means clustering method to adjust the preselected box, effectively improving the recognition accuracy but slowing down the detection speed. Fu et al. (2020) used the Faster-RCNN to identify apples. Before model establishment, a depth filter was used to remove the background of fruit trees in the image, improving recognition accuracy by 2.5% compared with the original network model. Tian et al. (2019) proposed an improved YOLO-V3 model to detect apples at different growth stages in orchards, and the average time of detection model was 0.304 s for images with 3000×3000 resolution. Li et al. (2021) identified occlusion and small object green pepper based on the deep learning object detection algorithm of Yolov4-tiny combining the attention mechanism and multi-scale prediction. The average precision value of the model reached 95.11%, the accuracy rate was 96.91%, and the recall rate was 93.85%. Zhang et al. (2021) proposed a recognition and positioning method for cherry tomatoes based on a lightweight neural network improved YoloV4-Lite, and the recognition accuracy and *AP* were improved by 8.29% and 0.15%, respectively, compared with the original network model. Xiong et al. (2020) added residual network to YOLOv3 model for night citrus recognition, and the recognition accuracy and the recognition speed was increased by 2.27% and 26%, respectively, compared with the original network model. Cecotti et al. (2020) used transfer learning to pre-train the network and data enhancement to increase the number of samples. They also used a modified Resnet network to identify grapes and perform yield estimates, which achieved good accuracy. Giang et al. (2022) rapidly detected tomatoes based on semantic segmentation neural network of RGB-D image, and the detection accuracy rate was 80.2%. Huang et al. (2022) applied the YOLOv5 algorithm to detect the citrus data set collected by UAV, and the detection accuracy rate was 93.32%. Yan et al. (2021) proposed a lightweight apple object detection method using improved YOLOv5s to identify grasping and ungrasping apples in apple tree images automatically, and the recognition recall rate, accuracy, *AP* and *F*_1_ were 91.48%, 83.83%, 86.75% and 87.49%, respectively. Zhang et al. (2022) applied the YOLOX object detection algorithm to carry out the counting detection of Holly fruit and tested the counting efficiency under different distances and scenarios. Zhou et al. (2022) proposed an enhanced YOLOX-s object detection algorithm. Compared with the original YOLOX-s, the enhanced model improved the detection *AP* of kiwifruit images by 6.52%, reduced the number of model parameters by 44.8% and upgraded the model detection speed by 63.9%. Miao et al. (2022) developed an efficient tomato picking robot based on traditional image processing methods and YOLOv5 object detection algorithm, which had high detection accuracy under different lighting conditions, with an average deviation of 2 mm and a picking time of 9 s/cluster. Wang et al. (2022) proposed an improved YOLOv4 model for pear detection in the natural environment. The *AP* of the model was 96.71%, the model size was reduced by approximately 80%, and the average detection speed was 0.027 s. Many researchers have researched fruit target detection based on CNN and achieved good results, but they mainly realised fruit detection under daytime conditions. During the growth of dragon fruits, supplemental light is carried out at night, providing an advantageous condition for the all-weather picking of dragon fruits. Few reports have focused on target detection for picking dragon fruits in all weather.

Thus, this study constructed a lightweight neural network model to reduce the size of the network model and improve the detection accuracy, which was used for the all-weather real-time detection task of dragon fruit picking robots in complex scenes. The main innovations and contributions are summarised as follows:

1. To establish data sets of dragon fruits under different lighting conditions. Through data enhancement, the image dataset was diversified, and the anti-interference ability under complex conditions was enhanced.
2. The lightweight ghost module was adopted in the model, replacing the conventional convolution of the original YOLOv5s network by combining a small number of convolution kernels and linear change operations to achieve the lightweight improvement of the model. The coordinate attention mechanism (CAM) was added to the original YOLOv5s network to make the model more accurate in locating and identifying dense dragon fruit. The feature fusion of different scales was strengthened by constructing a bidirectional feature pyramid network (BiFPN). The SIoU loss function was used to replace the original loss function to improve the convergence speed during model training.

The rest of the paper was structurally organised as follows: The second section presents the data material, including dragon fruit growth characteristics, image acquisition and dataset construction. The third section introduces the improved YOLOv5s dragon fruit detection model, which mainly includes the lightweight improvement of the model, the introduction of CAM and BiFPN, and the improvement of the loss function. The fourth section introduces the training and testing of the model, including the training platform information, parameter setting of the training network and evaluation index. The fifth section presents the test results and discussion. The final section illustrates the conclusions and prospects of the study.

## 2 Data materials

### 2.1 Growth characteristics of dragon fruit

**Figure 1:**
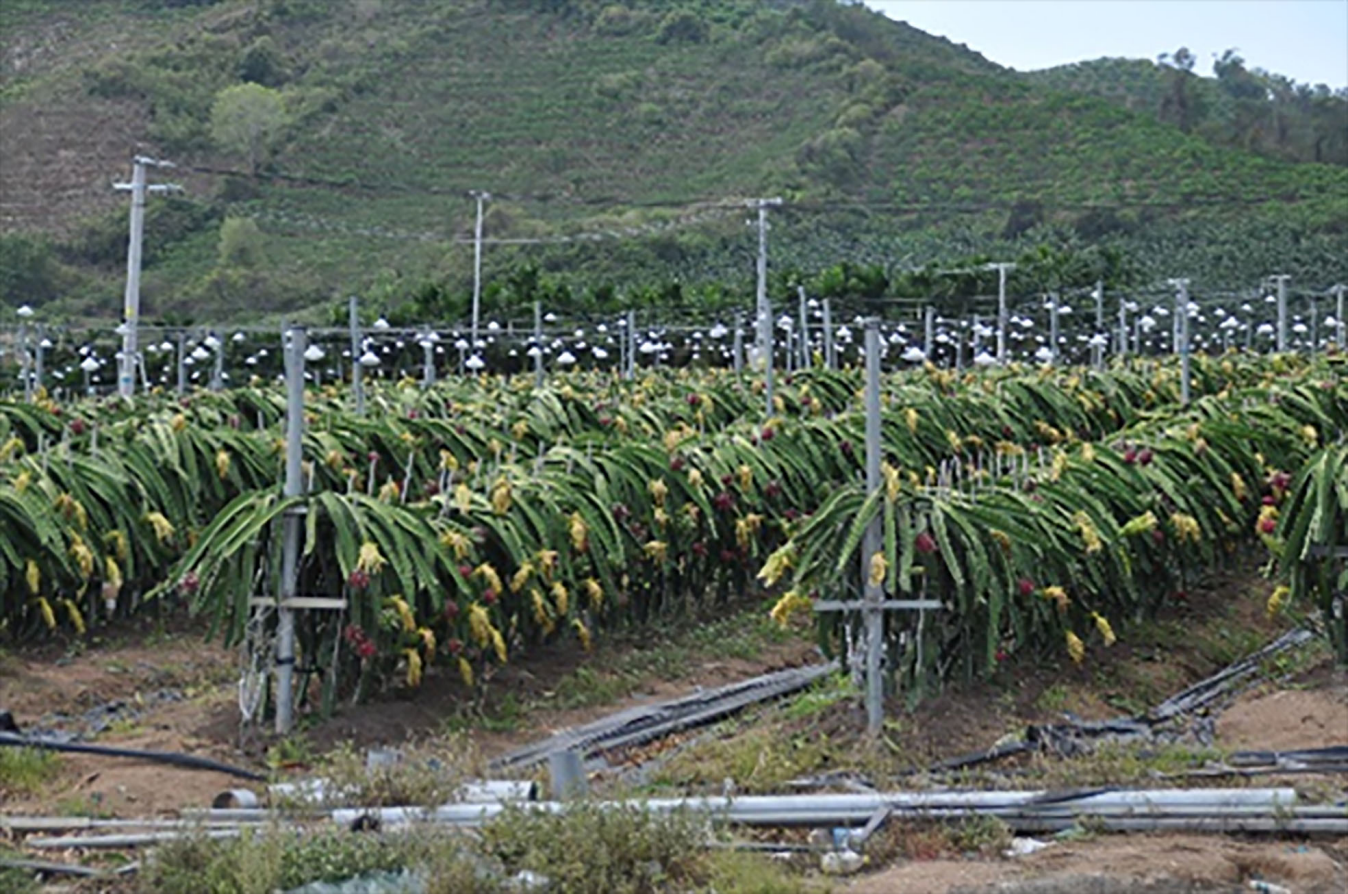
Planting pattern of dragon fruit.

Dragon fruit is a plant of the cactus family. Its branches are mostly triangular, and its edge width is generally 3–8 cm. It has many branches and is mainly cultivated by dense trellis planting (**Figure 1**). As a typical tropical and subtropical fruit, the shape of dragon fruit is generally spherical, the length of the fruit is 7–12 cm, and the diameter of the fruit is 5–10 cm. Fruits of the dragon fruit are distributed on branches. Given that fruits are blocked by branches and overlap with each other, accurately identifying dragon fruit, counting and measuring production, monitoring fruit diseases and insect pests and accurately picking fruit using picking robots in the field are difficult.

### 2.2 Image acquisition

**Figure 2:**
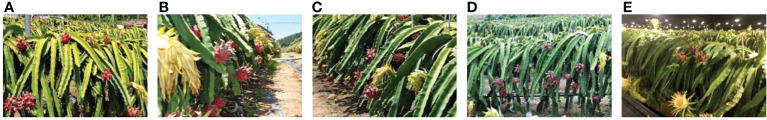
Dragon fruit images under different lighting conditions. **(A)** Front light. **(B)** Back light. **(C)** Side light. **(D)** Cloudy day. **(E)** Night.

This study took the red dragon fruit cultivated by dense trellis planting in modern standard orchards as the research object. The dragon fruit images were collected from the dragon fruit planting base in Yazhou, Sanya City, Hainan Province (latitude: 18.20.45, longitude: 109.12.14). Nikon SLR cameras and intelligent mobile phones were used to collect images of dragon fruits. Multi-scale dragon images were acquired during the three periods of sunny day, cloudy day and night. In the shooting process, the operation process of the picking robot was simulated, and the shooting angle and distance were constantly changed. A total of 1987 images were collected. Among them, shooting conditions on sunny days included front light, side light and back light. The collected images included varying maturity, attitude, size, lighting, background and fruit overlap occlusion. The image resolution is 4,288×2,848 pixels, and the format is JPEG. **Figure 2** shows the dragon fruit images collected under different lighting conditions.

### 2.3 Construction of data set

The collected data were clipped and compressed to 640×640 pixels to improve the training efficiency of the network model and shorten the training time in the training stage of the deep learning model. The LabelImg annotation tool was used to annotate the rectangular box of the dragon fruit in the image manually. During annotation, all mature dragon fruits fully exposed in the image were labelled in a rectangular frame, the exposed part of overlapping or occluded mature dragon fruits were labelled, and the mature dragon fruits with occlusion degrees less than 5% in the image were not labelled. A total of 1,987 images were labelled, and the number of labelled mature dragon fruits was 5,123. After annotation, the.xml file containing the ground truth was obtained. To avoid the phenomenon of sample imbalance and overfitting of model training, data enhancement technology was used to expand the size of dragon fruit data sets and improve the robustness and generalisation ability of the model. Under different lighting conditions, for the original dragon fruit images with obvious features, defocus blur, motion blur, pixelation and cloud were used to enrich the data features, increase the number of training data and reduce the unbalanced proportion of samples and the sensitivity of the model to the image to improve the model robustness. **Table 1** shows the basic information of the specifically constructed dragon fruit data sets.

**Table 1:** Basic information of dragon fruit data sets.

	Daytime					
Category	Sunny day			Cloudy day	Night	Sum
	Front light	Back light	Side light			
The number of original image	390	310	440	347	500	1987
The number of enhancement of data	1000	1000	1000	1000	1000	5000
The number of marked dragon fruit	2930	2814	3070	3035	2883	14732

The constructed dragon fruit data sets were divided into the training and testing sets according to the ratio of 8:2, and the number of dragon fruit image samples in the train and test sets was 4,000 and 1,000, respectively. There were no duplicate images between the training set and testing set.

## 3 Improvement of detection model for dragon fruit based on YOLOv5s

### 3.1 The network structure of YOLOv5s

**Figure 3:**
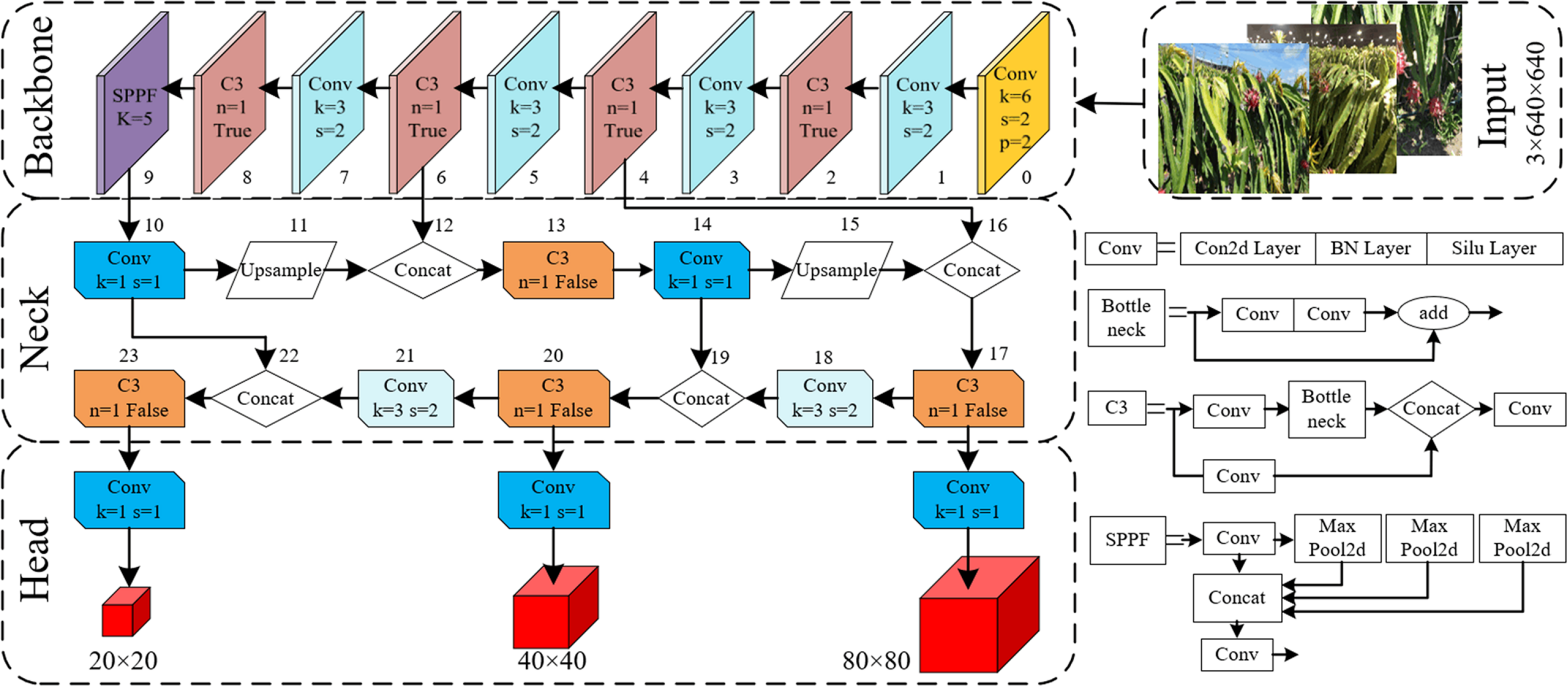
Network structure diagram of YOLO V5 model.

The network structure of the YOLOv5s model is a classical one-stage structure, as shown in **Figure 3**, which is composed of four parts: input, backbone, neck and prediction head. Mosaic data enhancement, adaptive anchor frame calculation, adaptive image scaling and other methods are used at the input. The backbone part integrates Conv, C3, SPPF and other feature extraction modules for feature extraction. The neck part adopts the PANet structure for multi-scale feature fusion to strengthen feature extraction and greatly improve the model effect. Compared with other Faster-RCNN, SSD and YOLO series models, this model has fewer parameters, a small weight file and the advantages of fast reasoning speed and high detection accuracy. Therefore, the detection model for dragon fruit was designed based on the YOLOv5s deep convolutional network, which was conducive to the embedded development of the dragon fruit picking robot vision system.

### 3.2 Improved YOLOv5s detection model for dragon fruit

Given the multi-scale and multi-mode characteristics of all-weather picking and recognition of dense trellis planting fruit in a natural environment, a lightweight neural network model with high recognition accuracy based on YOLOv5s network structure was proposed, which is suitable for all-weather real-time detection task of dragon fruit picking robot in complex scenes. Firstly, the lightweight ghost module was used to replace the conventional convolution of the original YOLOv5s backbone network by combining a small number of convolution kernels and linear change operations, which effectively realised the lightweight improvement of the YOLOv5s network model. Secondly, CAM was added to the original YOLOv5s network, which could capture the cross-channel information and the information of direction perception and position perception, so that the model could accurately locate and identify the dense dragon fruit. Thirdly, the PANet feature fusion network was improved, and the BiFPN was built to enhance the transmission of feature information between different network layers, realise two-way feature fusion of deep and shallow layers and improve the detection effect of dragon fruit at different scales. Finally, the SIoU loss function was used to replace the original loss function to improve the convergence speed of model training.

#### 3.2.1 Network lightweight improvement

Ghost module is a method to realise a lightweight neural network (Han et al., 2020), which can make the deep neural network transplant the network to some mobile devices with relatively weak computing power on the basis of ensuring the performance ability of the algorithm. The overall direction is to reduce the number of network model Params and FLOPs.

**Figure 4:**
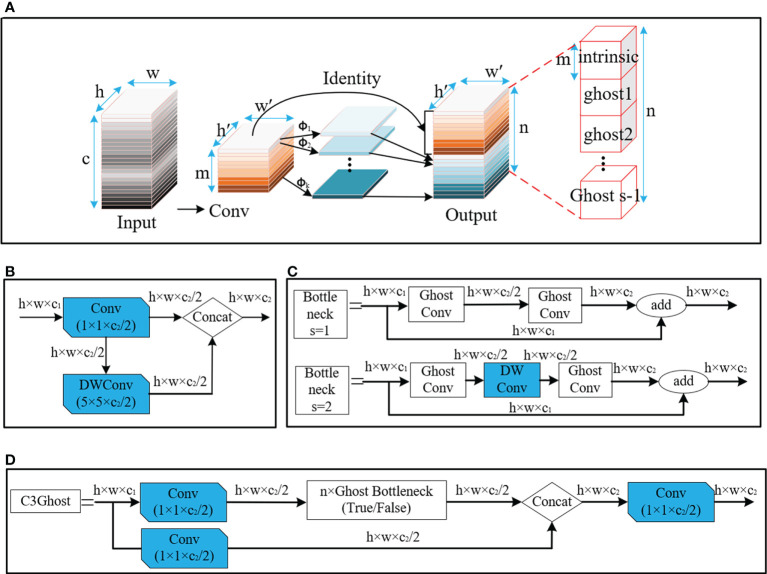
Structure of ghost. **(A)** Diagram of ghost module. **(B)** Ghost Conv. **(C)** GhostBottleneck. **(D)** C3 Ghost.

As shown in **Figure 4**, the ghost module uses a simple linear operation Φ instead of the original convolution operation to generate ghost graphs. Suppose that the size of the input feature graph is h × w × c convolved with n sets of convolution kernels of size k×k, and the size of the output feature graph is h′×w′×n. In the ghost model, m groups of k×k kernels are convolved with input to generate the intrinsic graph intrinsic of m×h′×w′, after which the intrinsic graph is linearly transformed Φ to produce the Ghost graph, and intrinsic and ghost together are used as output. Compared with ordinary convolution, after the ghost module is adopted, the model acceleration ratio *r_s_* and compression ratio *r_c_* are obtained, as shown in Equations (1) and (2).


(1) $$r_{s}=\frac{n\cdot h'\cdot w'\cdot c\cdot k\cdot k}{\frac{n}{s}\cdot h'\cdot w'\cdot c\cdot k\cdot k+\left(s-1\right)\cdot \frac{n}{s}\cdot h'\cdot w'\cdot c\cdot d\cdot d}\approx s$$

(2) $$r_{c}=\frac{n\cdot c\cdot k\cdot k}{\frac{n}{s}\cdot c\cdot k\cdot k+\left(s-1\right)\cdot \frac{n}{s}\cdot c\cdot d\cdot d}\approx s$$

These equations reveal that, compared with ordinary convolution, the ghost module reduced the calculation amount and the number of parameters in the convolution process to a certain extent. A large number of Conv and C3 modules in the original YOLOv5 network model are found, resulting in a large calculation amount and parameter volume of the model. The lightweight improvement of the network model is completed by using the ghost module to replace the Conv modules of layers 1, 3, 5, 7, 10, 14, 18 and 21 of the original network model with GhostConv and the C3 modules of layers 2, 4, 6 and 8 with C3Ghost module for calculation.

#### 3.2.2 Coordinate attention mechanism (CAM)

**Figure 5:**
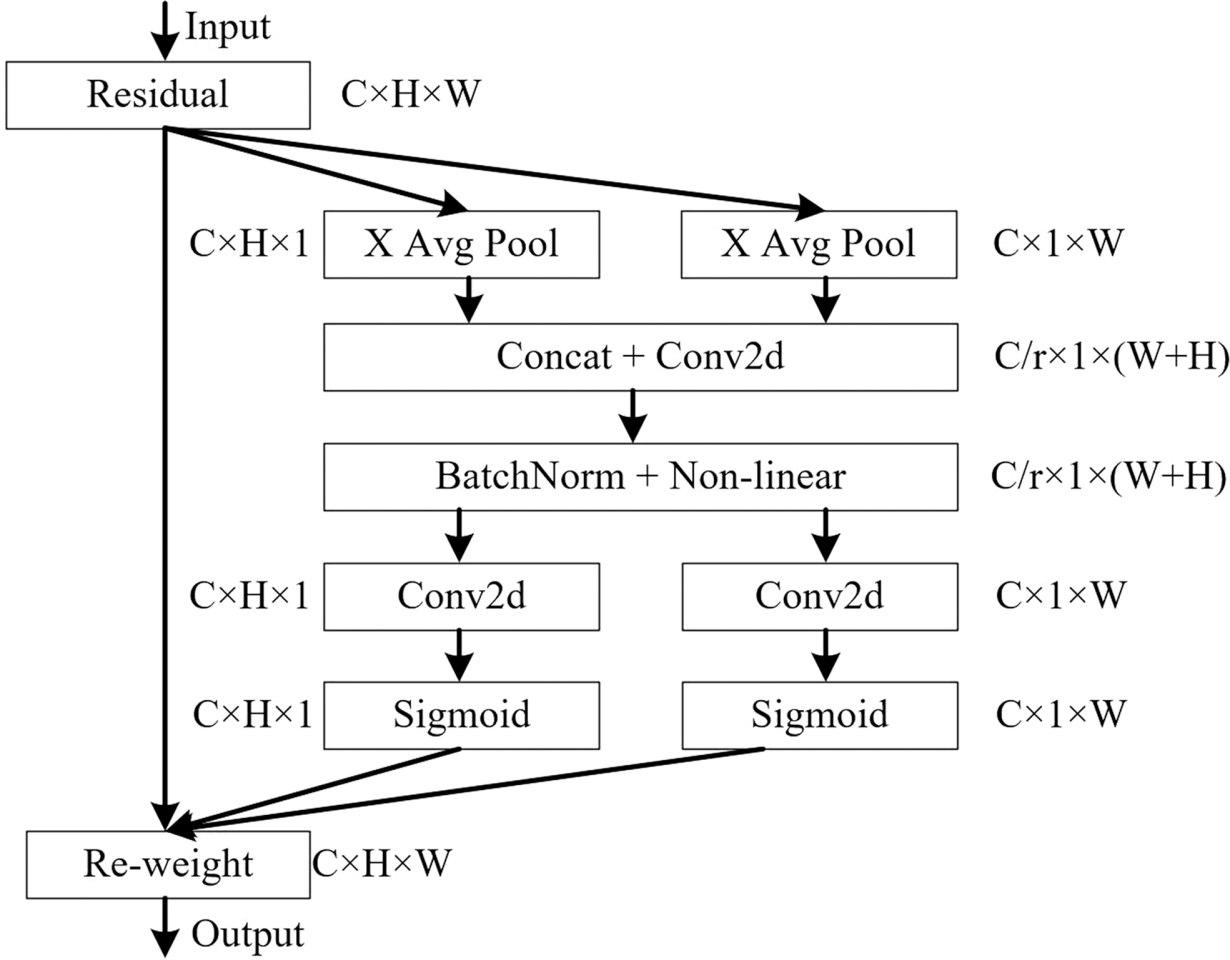
Structure diagram of Coordinate Attention module.

The detection of small dense objects in densely planted dragon fruit orchards is easily influenced by different lighting conditions, especially in the night scene, when the detection is difficult. The original YOLOv5 network model easily loses the feature information of dense objects and small objects in the reasoning process, and the detection effect of small dense objects is poor. As shown in **Figure 5**, CAM is a novel mobile network attention mechanism proposed by embedding location information into channel attention (Hou et al., 2021). To alleviate the problem of location information loss caused by two-dimensional global pooling proposed by previous attention mechanisms, such as SENet (Hu et al., 2018) and CBAM (Woo et al., 2018), CAM decomposed channel attention into two one-dimensional feature coding processes, which aggregated features along two spatial directions respectively.

A CAM block can be viewed as a computational unit that can take any intermediate feature tensor *X*=[*x*_1_,*x*_2_,⋯,*x*_*c*_]∈*R* ^*C*×*H*×*W*^ as input and output with the same size as the intermediate feature tensor *Y*=[*y*_1_,*y*_2_,⋯,*y*_*c*_]. Meanwhile, it has the effect of enhancing representation.

Therefore, CAM was inserted in this study after layers 4, 6, 8, 9, 17, 20 and 23 of the original YOLOv5 network model. After data enhancement, the dragon fruit images entered the main network for feature extraction and then entered the neck part of the model through the CAM connected between the main part and the neck part (layers 4, 6 and 9 of the original network). In the neck part, the dragon fruit image feature fusion of different scales was carried out. Finally, CAM, after the 17th, 20th and 23rd layers of the original network, entered the prediction head part of the model, so that the network model can more accurately notice the dense small dragon fruit objects. It improved the detection ability of the network.

#### 3.2.3 Construction of the bidirectional feature pyramid network (BiFPN)

**Figure 6:**
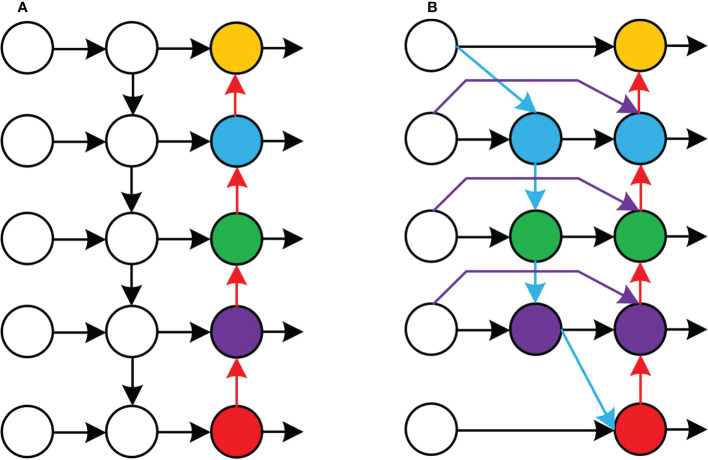
Structures of PANet and BiFPN. **(A)** Structure of PANet. **(B)** Structure of BiFPN.

The neck part of the original YOLOv5 network model uses PANet for multi-scale feature fusion, and the three effective feature layers of different scales obtained in the backbone part continue to extract features in the neck part. When fusing different input features, PANet adds the features without distinction. However, because these different input features have different resolutions, their contributions to the fused output features tend to be unequal. BiFPN is a new feature fusion method proposed by the Google Brain team, which realises the two-way fusion of top-down and bottom-up deep and shallow features and enhances the transmission of feature information between different network layers. The PANet and BiFPN structures are shown in **Figure 6**.

In this study, the PANet in YOLOv5 structure is replaced by BiFPN, and the Concat of layer 16, 20, 24 and 28 in the network structure is renamed as BiFPN_Concat. To fuse more image features without consuming more computational cost, the image features output from the 8th layer network were fused to the 24th layer network by concatenation, and the image features output from the 11th layer network are fused to the 28th layer network by concatenation, so as to achieve a higher level of feature fusion. BiFPN used the fast normalised fusion, which is normalised by dividing the use-right value by the sum of the ownership value. It normalises the weights to between 0 and 1 to improve the detection speed.

#### 3.2.4 Improvement of loss function

The traditional object detection loss function relies on the aggregation of bounding box regression indicators, such as the distance, overlap region and aspect ratio of the predicted box and real box (i.e. GIoU, DIoU and CIoU). The original YOLOv5 network model used the CIoU loss function, but it did not consider that the situation of required direction does not match between the real box and predict box. It led to a slower and less efficient convergence of the network model during training. At the same time, the predicted box may “wander around” during training and produce worse models. To solve the above problems, SIoU loss function was used to replace the original loss function, which could introduce the vector angle between the real box and the predicted box (Gevorgyan, 2022). It includes four parts: angle cost, distance cost, shape cost and IoU cost.

**Figure 7:**
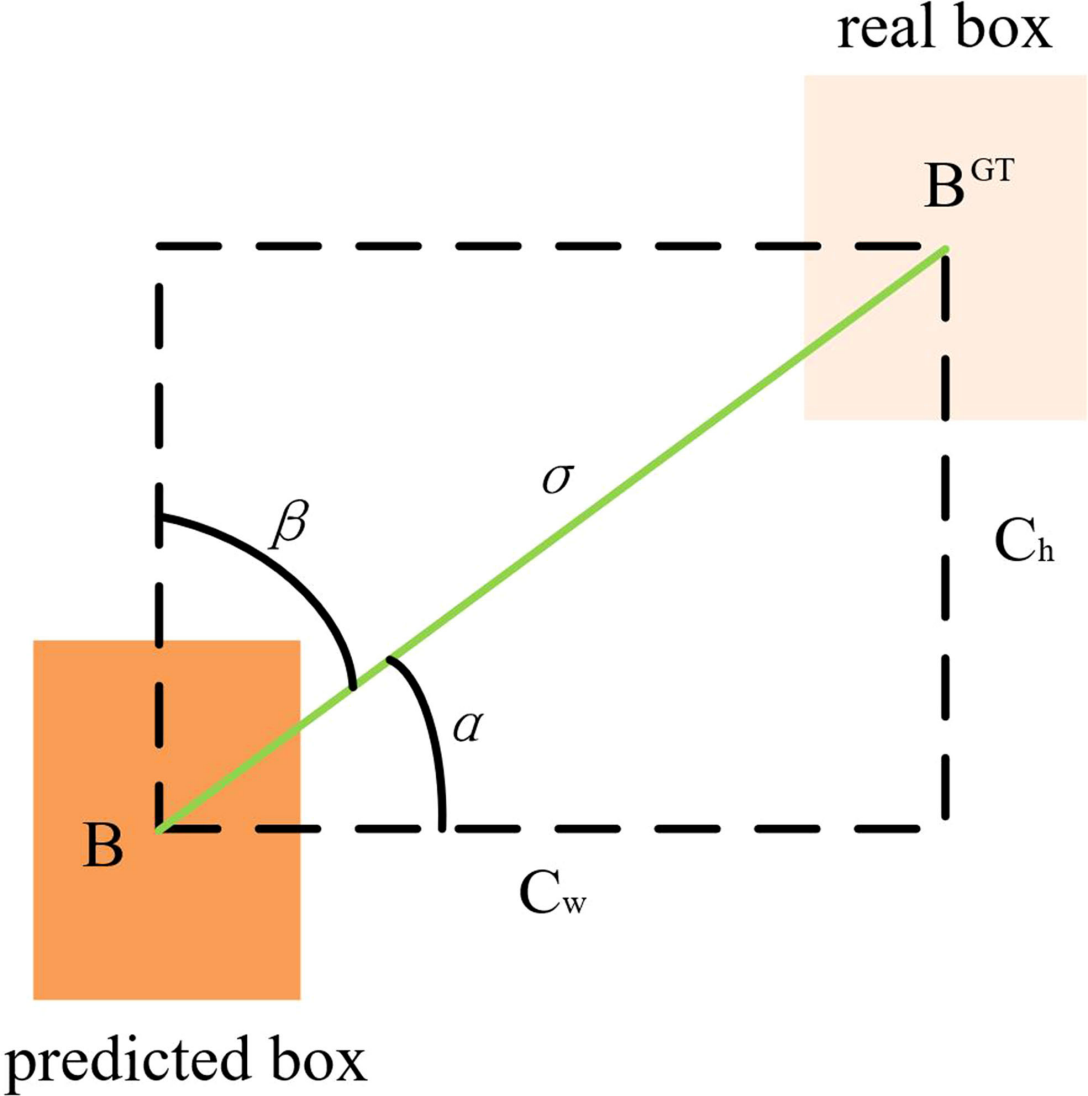
The scheme for calculating the contribution of the Angle cost to the loss function.

Angle cost is shown in Equation (3). **Figure 7** shows that, when *α* is π/2 or 0, the angle cost is 0. In the training process, if *α* is less than π/4, *α* is minimised; otherwise, *β* is minimised.


(3) $$\Lambda =\text{cos}[2\times {\text{sin}}^{2}(\text{arcsin}\frac{c_{h}}{\sigma }-\frac{\pi }{4})]$$

Among them,


(4) $$\sigma =\sqrt{{\left(b_{c_{x}}^{gt}-b_{c_{x}}\right)}^{2}+{\left(b_{c_{y}}^{gt}-b_{c_{y}}\right)}^{2}}$$

(5) $$c_{h}{\text{=max(b}}_{c_{y}}^{gt},{\text{b}}_{c_{y}})-{\text{min(b}}_{c_{y}}^{gt},{\text{b}}_{c_{y}})$$

where, *c_h_* is the height difference between the centre points of the real box and the predicted box, *σ* is the distance between the centre points of the real box and the predicted box, $\left(b_{c_{x}}^{gt},b_{c_{y}}^{gt}\right)$ is the centre coordinates of the real box and (*b*_*c*_*x*_ _,*b*_*c*_*y*__) is the centre coordinates of the predicted box.

Distance cost Δ is shown in Equation (6),


(6) $$\Delta =2-e^{-(2-\Lambda ){\left(\frac{b_{c_{x}}^{gt}-b_{c_{x}}}{c_{w1}}\right)}^{2}}-e^{-(2-\Lambda ){\left(\frac{b_{c_{y}}^{gt}-b_{c_{y}}}{c_{h1}}\right)}^{2}}$$

where, (*c_w_*_1_, *c_h_*_1_) is the width and height of the minimum outer rectangle of the real box and the predicted box.

The shape cost Ω is shown in Equation (7),


(7) $$\Omega =(1-e^{-\frac{\left|w-w^{gt}\right|}{\max(w,w^{gt})}}{)}^{\theta }+(1-e^{-\frac{\left|h-h^{gt}\right|}{\max(h,h^{gt})}}{)}^{\theta }$$

where, (*w*, *h*) is the width and height of the predicted box, (*w^gt^*, *h^gt^*) is the width and height of the real box and *θ* is the degree of attention to shape loss.

To sum up, the SIoU loss function is defined as Equation (8).


(8) $${\text{Loss}}_{\text{SIoU}}=1-\text{IoU}+\frac{\Delta +\Omega }{2}$$

#### 3.2.5 Improved detection model for dragon fruit

The overall structure of the improved detection model network for dragon fruit is shown in **Table 2**. The from column in the table indicates which layer the input comes from, −1 represents the output from the previous layer, −2 represents the output from the upper layer. The params column represents the size of the argument, module is the name of the module, and the arguments are the information about the module argument, including the number of input channels and output channels, the size of the convolution kernel and the step size information.

**Table 2:** The overall structure of the improved network.

Number	From	Params	Module	Arguments
0	-1	3520	Conv	[3, 32, 6, 2, 2]
1	-1	10144	Ghost Conv	[32, 64, 3, 2]
2	-1	12072	C3Ghost	[64, 64, 3]
3	-1	38720	Ghost Conv	[64, 128, 3, 2]
4	-1	47040	C3Ghost	[128, 128, 4]
5	-1	6704	CAM	[128, 128]
6	-2	151168	Ghost Conv	[128, 256, 3, 2]
7	-1	186976	C3Ghost	[256, 256, 5]
8	-1	13360	CAM	[256, 256]
9	-2	597248	Ghost Conv	[256, 512, 3, 2]
10	-1	679680	C3Ghost	[512, 512, 4]
11	-1	51296	CAM	[512, 512]
12	-1	656896	SPPF	[512, 512, 5]
13	-1	51296	CAM	[512, 512]
14	-1	69248	Ghost Conv	[512, 256, 1, 1]
15	-1	0	Upsample	[None, 2, ‘nearest’]
16	[-1,8]	2	BiFPN	[1]
17	-1	361984	C3	[512, 256, 1, False]
18	-1	18240	Ghost Conv	[256, 128, 1, 1]
19	-1	0	Upsample	[None, 2, ‘nearest’]
20	[-1,5]	2	BiFPN	[1]
21	-1	90886	C3	[256, 128, 1, False]
22	-1	6704	CAM	[128, 128]
23	-2	75584	Ghost Conv	[128, 128, 3, 2]
24	[-1,16,8]	3	BiFPN	[1]
25	-1	460288	C3	[896, 256, 1, False]
26	-1	13360	CAM	[256, 256]
27	-2	298624	Ghost Conv	[256, 256, 3, 2]
28	[-1,14,11]	3	BiFPN	[1]
29	-1	1444864	C3	[1024, 512, 1, False]
30	-1	51296	CAM	[512, 512]
31	[22,26,30]	26970	Detect	[nc, anchors]

## 4 Model training and testing

### 4.1 Training processing platform

This study built a deep learning framework based on PyTorch 1.7.1 to train and test the dragon fruit detection model. The relevant configurations of the test platform are as follows: Intel(R) Core(TM) i9-10900X CPU and NVIDIA GeForce RTX 3090 GPU (Dual Cards). The operating system is Windows 10. The acceleration environment is CUDA 11.3 and CUDNN 8.2.0. The development environment is PyCharm 2021.2.2 and Python 3.7. Other Python libraries are Numpy 1.21.6 and Opencv 4.6.0. The model input image size is 640×640 pixels. The training parameters are as follows: a batch size of 64, 300 training iterations, momentum of 0.937, initial learning rate of 0.001, attenuation coefficient of 0.9.

### 4.2 Evaluation indicators

This study used precision (*P*) to measure the accuracy of dragon fruit prediction. Recall (*R*) measures the detection of positive samples in all dragon fruit. Average precision (*AP*) measures the performance of the detector in each category. Mean average precision (*mAP*) is the average of all class *AP*s. *P*, *R*, *AP* and *mAP* are defined as (9)–(12). The complexity of the algorithm or model is measured by the number of parameters (Params) and floating-point operations (FLOPs).


(9) $$P=\frac{\text{TP}}{\text{TP}+\text{FP}}\times 100\%$$

(10) $$R=\frac{\text{TP}}{\text{TP}+\text{FN}}\times 100\%$$

(11) $$AP=\int_{0}^{1}P(R)dR\times 100\%$$

(12) $$mAP=\frac{\sum_{i=1}^{k}AP}{k}$$

where, TP is the number of correctly predicted positive samples, TN is the number of correctly predicted negative samples, FP is the number of negative samples divided into positive samples, FP is the number of positive samples divided into negative samples, and *k* is the number of categories.

## 5 Results and discussion

**Figure 8:**
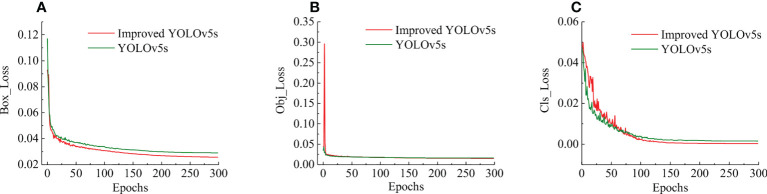
Comparison of Loss curves for model training. **(A)** The positioning loss curve. **(B)** The confidence loss curve. **(C)** The classification loss curve.

The training process of the original YOLOv5s model and the improved YOLOv5s model used the same data set and the same parameters. According to the log files saved in the training process, the training loss curves of the two models were drawn, as shown in **Figure 8**.

**Figure 8A** is the positioning loss curve, which was used to represent the error between the predicted box and the labelled box. After 10 rounds of iteration, the decline rate of positioning loss value started to become gentle. After 200 rounds of iteration, the positioning error tended to the stable state. At this point, the localisation loss of the improved YOLOv5s model was reduced by 0.01 compared with the original YOLOv5s. The model’s performance improved after the SIoU loss function was adopted. **Figure 8B** is the confidence loss curve, which calculates the network’s confidence in the iterative process. The confidence loss curves of the two models were consistent before and after the improvement. **Figure 8C** is the classification loss curve, which is used to show whether the aiming frame and the corresponding calibration classification are correct. After 100 rounds of iteration, the classification error of the model tended to the stable state, where the classification error of the improved YOLOv5s model was significantly reduced compared with the original YOLOv5s. **Figure 8** shows that, compared with the original YOLOv5s model, the improved YOLOv5s model has faster convergence and smaller loss value. The results showed that the convergence ability of the network was improved after modifying the original loss function.

### 5.1 Comparison of different algorithms

To compare the accuracy of different models in dragon fruit detection, eight representative network models of YOLOv3, YOLOv3-Tiny, YOLOv4-CSP, YOLOv4-Tiny, YOLOv5s, YOLOX-s, YOLOv7 and YOLOv7-Tiny, were selected to compare and test with the improved YOLOv5s. All models used the same data set of dragon fruit for training and testing. The value of *mAP*, *P*, *R*, model size, Params and FLOPs were selected as model evaluation indicators. **Table 3** shows the detection results of dragon fruit for a different model.

**Table 3:** Identification results of dragon fruit for different model.

Model	*mAP*/%	*P*/%	*R*/%	Model size/MB	Params/M	FLOPs/G
YOLOv3	97.1	96.0	94.6	123.6	58.7	155.3
YOLOv3-Tiny	95.3	93.0	91.8	17.5	8.3	13.0
YOLOv4-CSP	94.3	91.6	87.3	105.5	50.1	119.7
YOLOv4-Tiny	92.9	92.3	88.1	6.3	2.9	6.4
YOLOv5s	96.3	94.7	93.7	14.5	6.4	15.8
YOLOX-s	93.5	90.1	86.5	34.3	9.0	26.7
YOLOv7	95.6	93.9	89.6	74.9	35.5	105.2
YOLOv7-Tiny	96.0	90.8	92.8	12.3	5.7	13.2
Improved YOLOv5s	97.4	96.4	95.2	11.5	5.2	11.4

**Table 3** shows that, compared with YOLOv3, YOLOv3-Tiny, YOLOv4-CSP, YOLOv4-Tiny, YOLOv5s, YOLOX-s, YOLOv7 and YOLOv7-Tiny, the improved YOLOv5s model has the highest *P*, *R* and *mAP* values of 96.4%, 95.2% and 97.4%, respectively. The model size, Params and FLOPs of the Improved YOLOv5s were larger than those of YOLOV4-Tiny, but they were smaller than other networks, which were 11.5 MB, 5.2 M and 11.4 G, respectively. Compared with the above eight network models, *mAP* of the improved YOLOv5s model was enhanced by 0.3%, 2.1%, 3.1%, 4.5%, 1.1%, 3.9%, 1.8% and 1.4% respectively, *P* was improved by 0.4%, 3.4%, 4.8%, 4.1%, 1.7%, 6.3%, 2.5% and 5.6% respectively, and *R* was improved by 0.6%, 3.4%, 7.9%, 7.1%, 1.5%, 8.7%, 5.6% and 2.4%, respectively. Compared with the lightweight YOLOV3-Tiny, YOLOv5s, YOLOX-s and YOLOV7-Tiny, the model size was reduced by 34.3%, 20.69%, 66.5% and 6.5%, Params was reduced by 37.3%, 18.7%, 42.2%, 8.8%, respectively, FLOPs was reduced by 12.3%, 27.8%, 57.3% and 13.6%, respectively. The results showed that the improved YOLOv5s model ensured good detection accuracy and realised the lightweight improvement of the network model. The improved YOLOv5s model could be embedded into the vision system of the dragon fruit picking robot to realise the automatic picking operation of dragon fruits.

### 5.2 Analysis of ablation experiment results

The ablation experiment is to verify the optimisation effect of each improved module, and the experimental results are shown in **Table 4**. Improved model 1 represents the introduction of the ghost module in the original network. Improved model 2 represents the modification of the pyramid structure of the original network. Improved model 3 represents the addition of CAM in the original network. Improved model 4 represents the modification of the loss function. Improved model 5 represents the addition of all the above improvement methods in the original network.

**Table 4:** Results of ablation experiment.

Model	Lightweight	Modifying the feature pyramid	Add attention mechanism	Modify the loss function	*mAP*/%	Params/M	FLOPs/G
YOLOv5s	×	×	×	×	96.3	6.4	15.8
Improved model 1	√	×	×	×	96.7	4.6	10.3
Improved model 2	×	√	×	×	97.2	6.8	16.2
Improved model 3	×	×	√	×	96.8	8.8	21.0
Improved model 4	×	×	×	√	96.8	6.4	15.8
Improved model 5	√	√	√	√	97.4	5.2	11.4

"√" Indicates that the current improvement method is used in the model, while "×" indicates that the current improvement method is not used in the model.

**Table 4** shows that, after the ghost module was used to lightweight the original YOLOv5s network structure, the Params was reduced by 28%, and the FLOPs was reduced by 34.8% compared with the original network model, but the *mAP* of the model increased by 0.4%. The main reason was that after the ghost module was used to replace the ordinary convolution in the original network, more feature maps were generated through linear operation, and this rich or even redundant information usually ensured a comprehensive understanding of the input dragon fruit features. Therefore, the lightweight network structure of the ghost module introduced into the original YOLOv5s network in this study could still ensure the detection accuracy of the model. When the CAM was added to the model, compared with the original model, the *mAP* of the model was improved by 0.5 percentage points, but the Params and the FLOPs of the model were increased by 2.4 M and 5.2 G, respectively. After replacing the PANet structure in the YOLOv5s network with BiFPN, the *mAP* of the model was improved by 0.9 percentage points, the Params and the FLOPs increased by 0.2 M and 0.7 G, respectively. After the loss function of the model was modified, the *mAP* of the model was improved by 0.5%. When these four improvements were combined into the model, compared with the original YOLOv5s network model, the *mAP* was increased by 1.1%, the Params was reduced by 18.7%, and the FLOPs was reduced by 27.8%. The results showed that improved YOLOv5s had better detection performance for dragon fruit objects, and the complexity of the model was reduced by using a lightweight module.

### 5.3 Analysis of detection results in different scenarios

To verify the feasibility of the improved YOLOv5s model, the dragon fruit images collected in different scenes were tested, including the scenes of front light, back light, side light, cloudy day and night. The results are shown in **Table 5**.

**Table 5:** Test results of dragon fruit recognition in different lighting scenes by the YOLOv5s model before and after improvement.

Model	*mAP*	*AP*					
		Front light	Back light	Side light	Cloudy day	Night
YOLOv5s	96.3	99.0	96.7	98.4	93.8	93.6
Improved YOLOv5s	97.4	99.5	97.3	98.5	95.5	96.1

**Table 5** shows that both models before and after improvement had the best recognition effect for dragon fruit in the scenes of the front light. The *AP* of the before and after improvement model in detecting dragon fruit under front light was 99.0% and 99.5%, respectively, and the *AP* of the improved model was improved by 0.5%. The *AP* of the before and after improvement model in detecting dragon under the backlight was 96.7% and 97.3%, respectively. The performance of the improved model was improved by 0.6%. The *AP* of the before and after improvement model in detecting dragon fruit under side light was 98.4% and 98.5%, respectively, and the performance of the improved model was improved by 0.1%. The *AP* of the model before and after the improvement under cloudy day was 93.8% and 95.5%, respectively, and the detection performance of the improved model was improved by 1.7%. The *AP* of the model before and after improvement at night is 93.6% and 96.1%, and the detection performance of the improved model is improved by 2.5%. According to **Table 5**, under different lighting conditions, the maximum deviation of the YOLOv5s model before improvement in detecting dragon fruit was 5.4%, and the maximum deviation after improvement was 3.4%, which was 2% lower than before the improvement. The improved model had the greatest improvement for the situation that was difficult to detect at night, indicating that the improved model was more robust to all-weather dragon fruit detection.

**Tables 6**, **7** show the detection visualisation results of the YOLOv5s model in different lighting scenes before and after the improvement. According to the visualisation results, the improved detection model had a better detection effect and stronger robustness in detecting dragon fruit objects of different scales under different lighting environments. The positioning was more accurate, and the model had a strong anti-interference ability in dense small object detection.

**Table 6:** Visual results of dragon fruit object detection on sunny days.

Front light	The original image	Visual result of YOLOv5s		Visual result of Improved YOLOv5s	
Single fruit big object	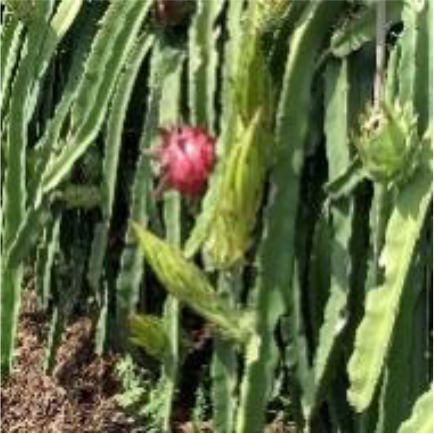	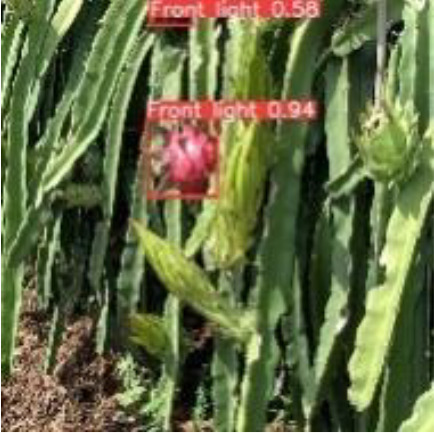	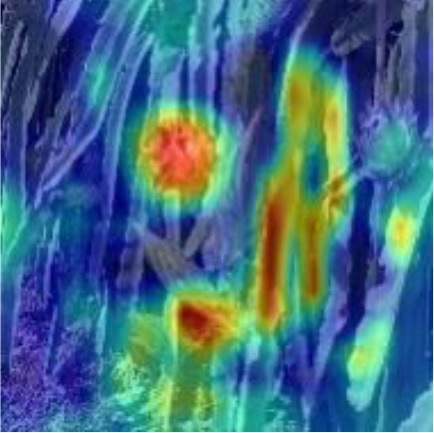	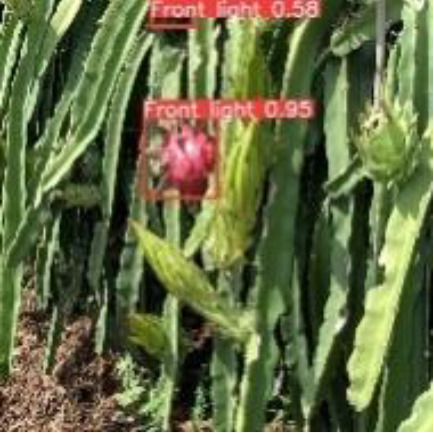	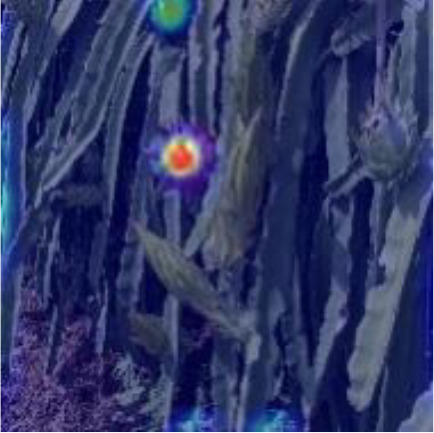
Many fruits in the goal	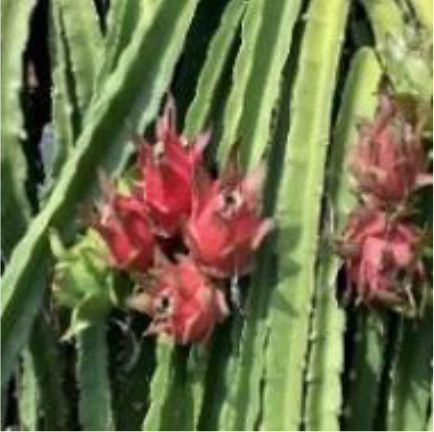	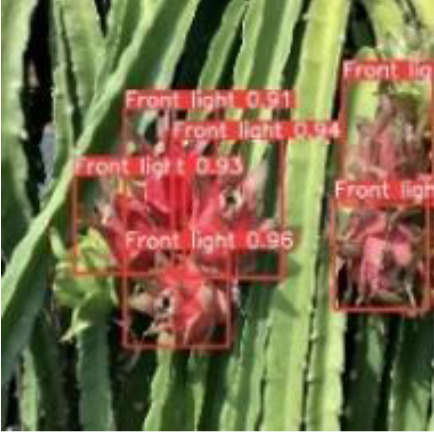	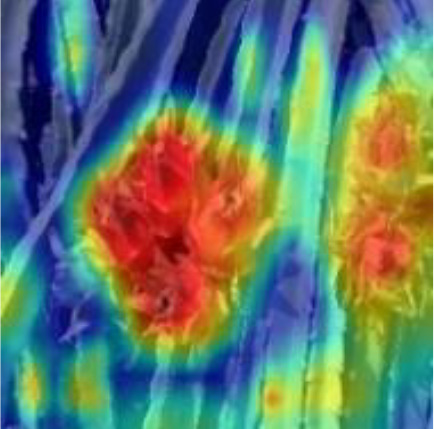	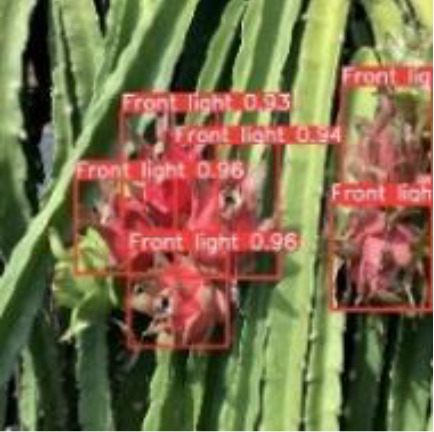	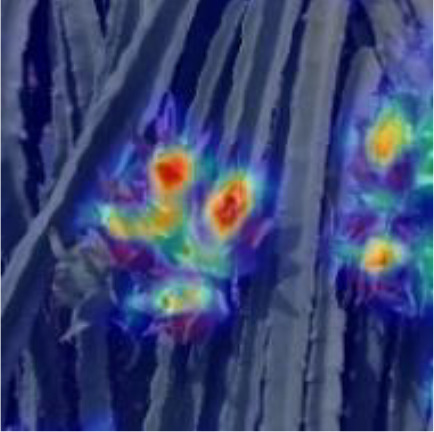
Dense small object	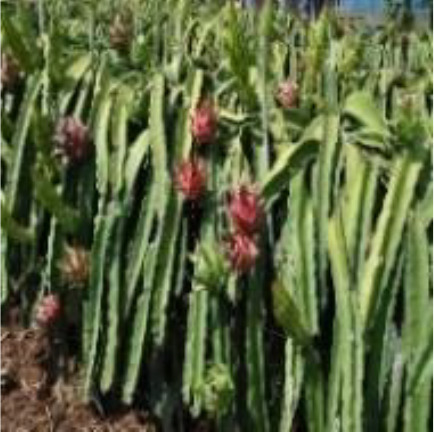	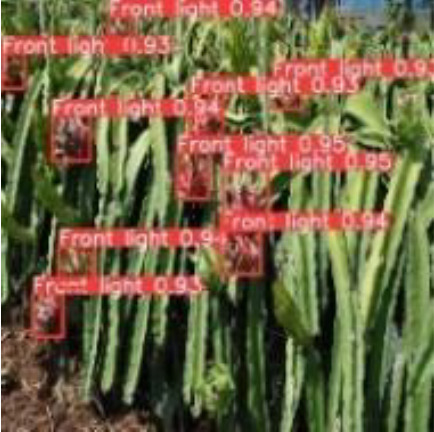	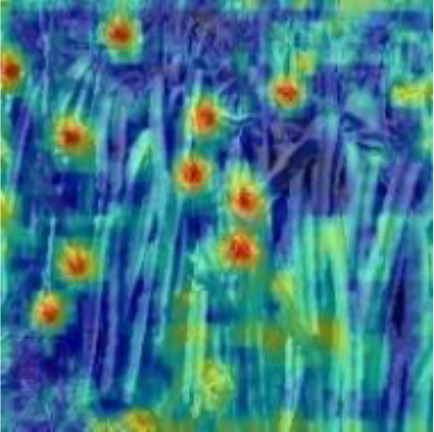	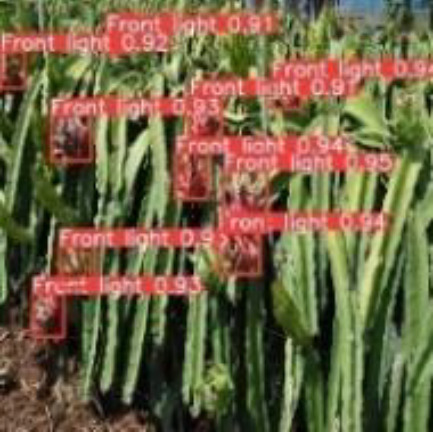	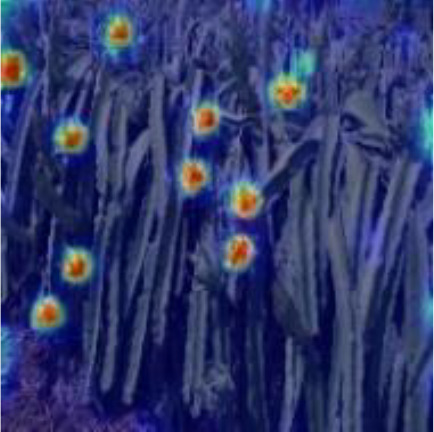
Back light	The original image	Visual result of YOLOv5s		Visual result of Improved YOLOv5s	
Single fruit big object	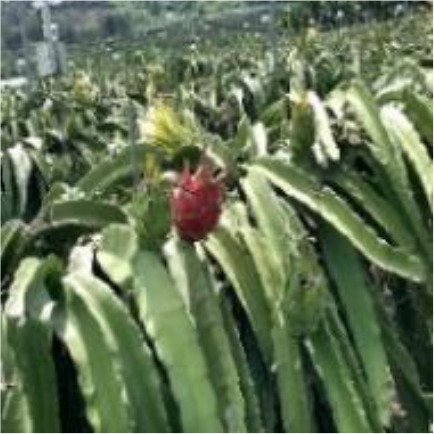	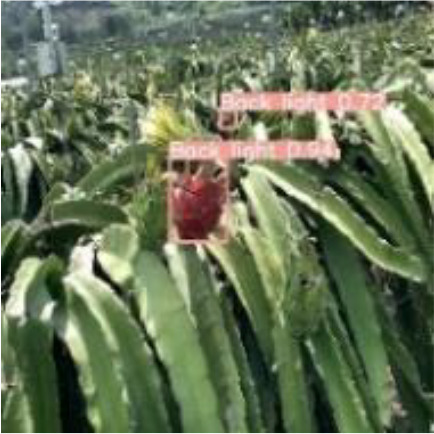	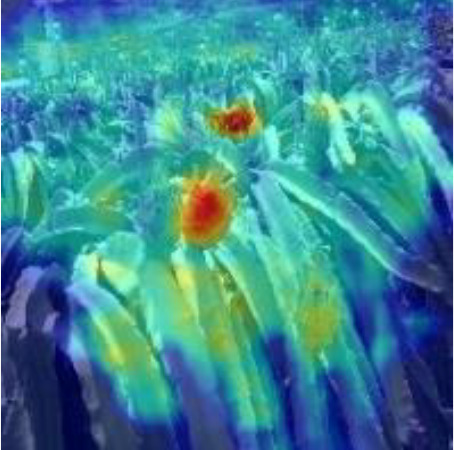	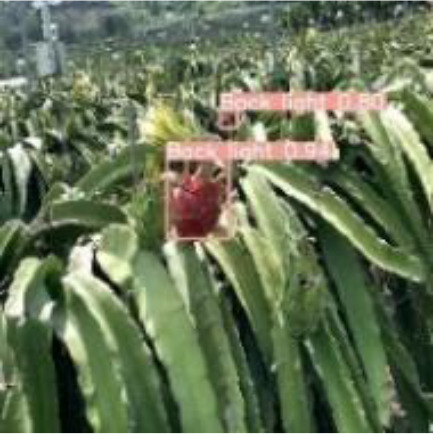	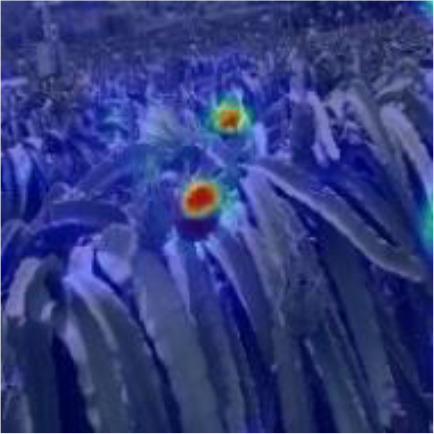
Many fruits in the goal	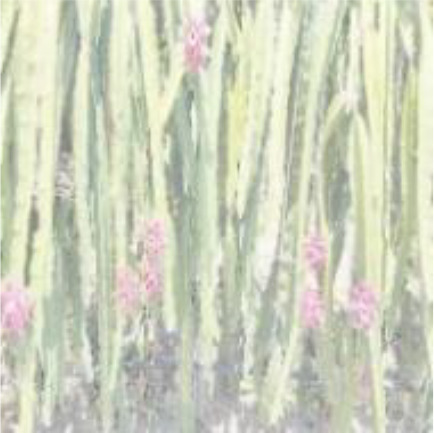	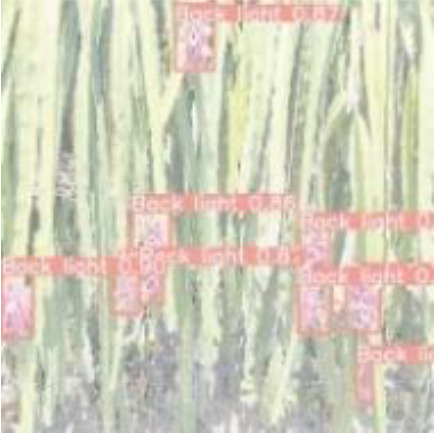	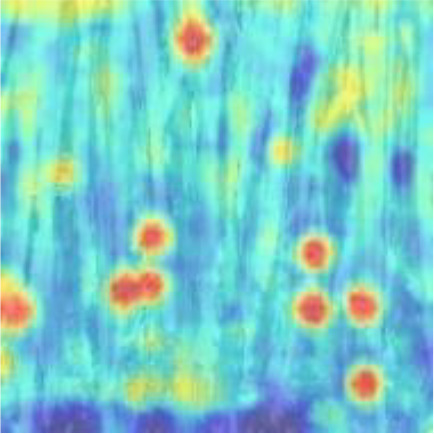	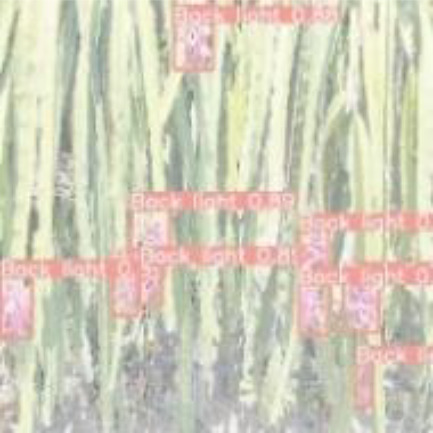	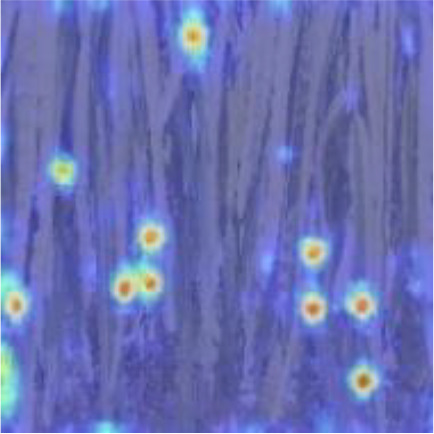
Dense small object	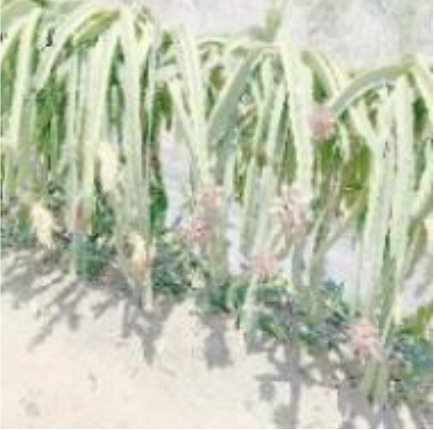	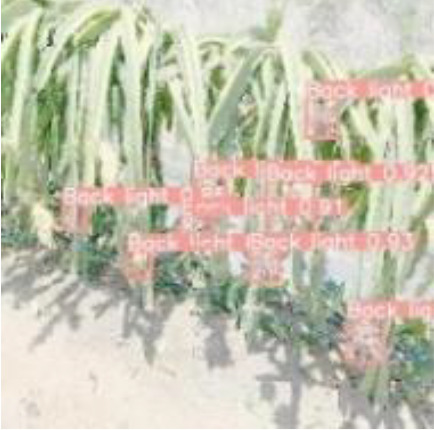	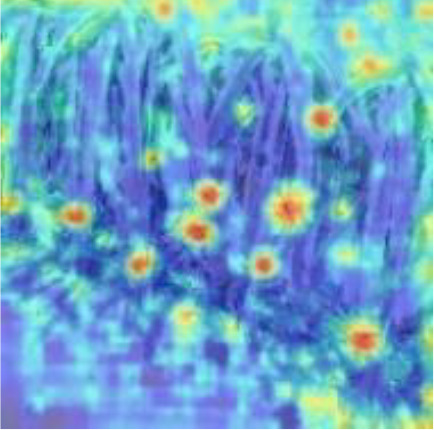	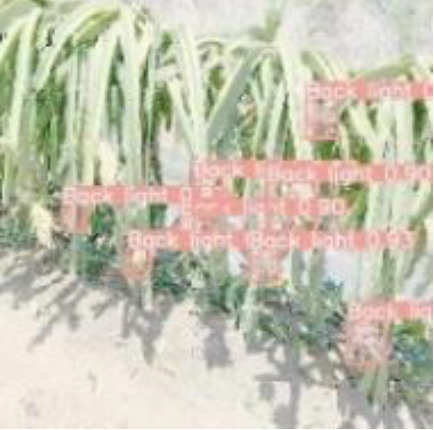	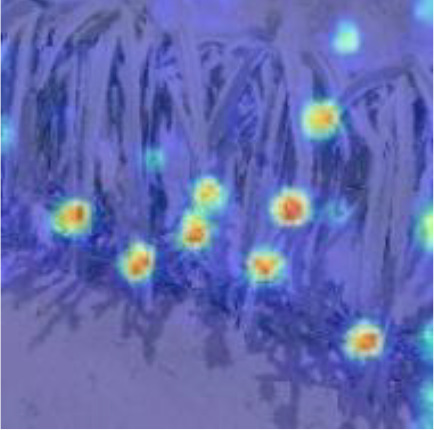
Side light	The original image	Visual result of YOLOv5s		Visual result of Improved YOLOv5s	
Single fruit big object	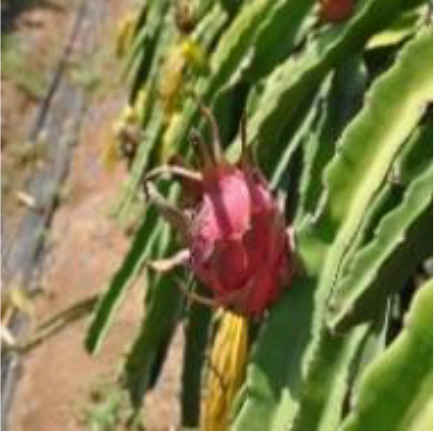	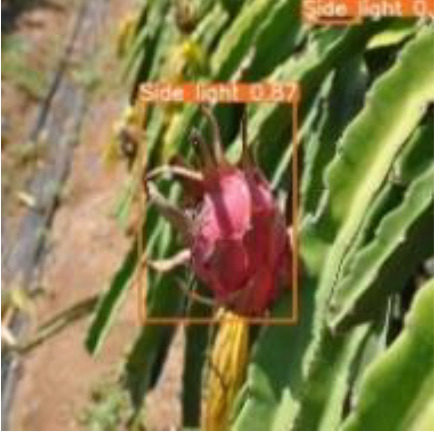	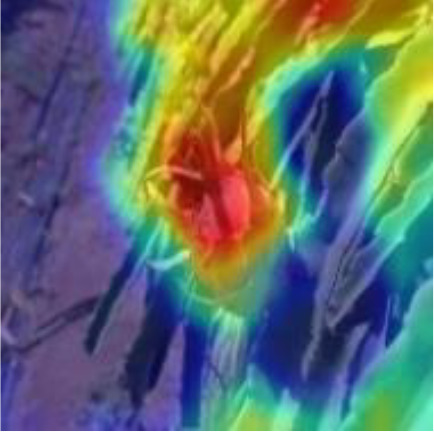	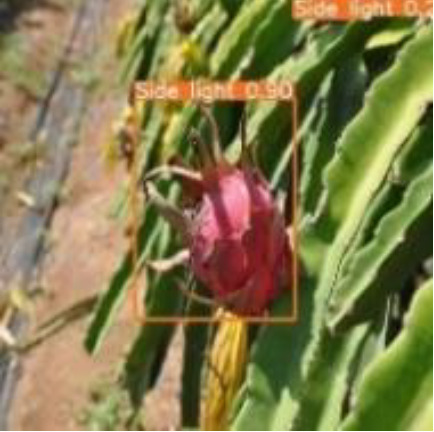	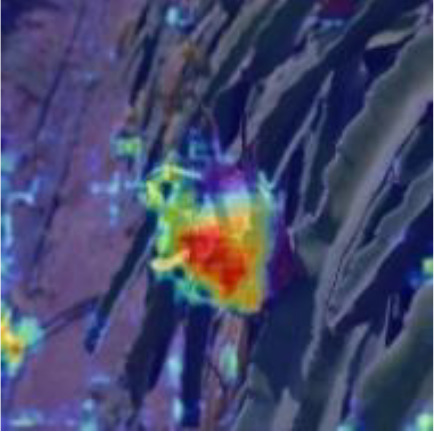
Many fruits in the goal	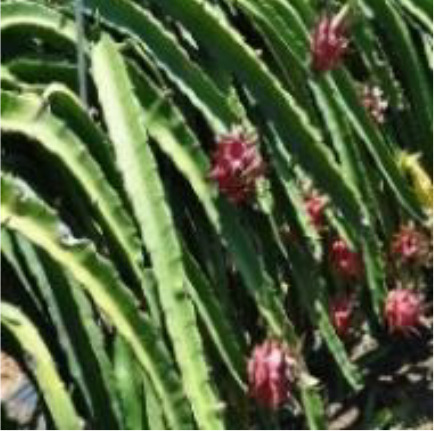	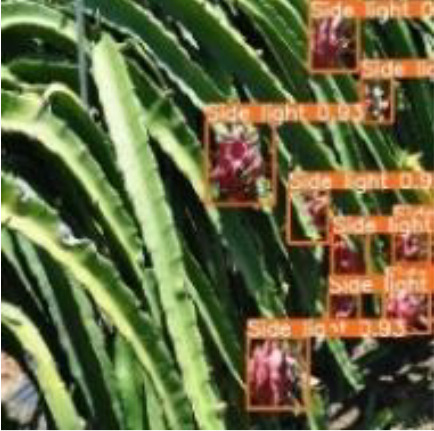	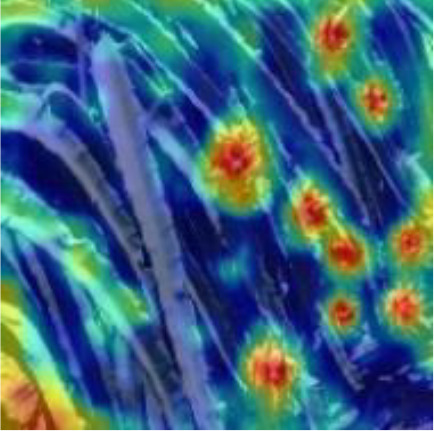	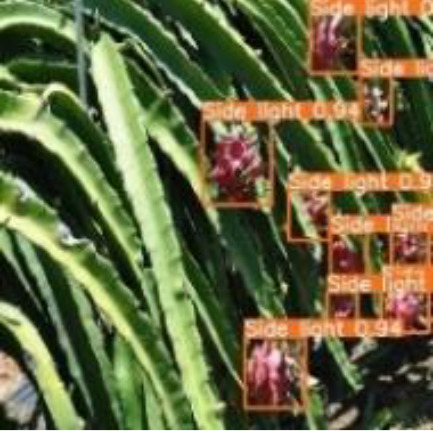	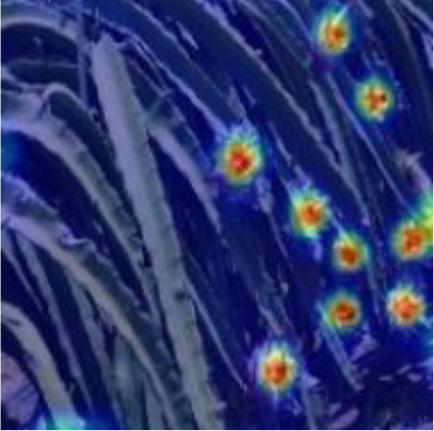
Dense small object	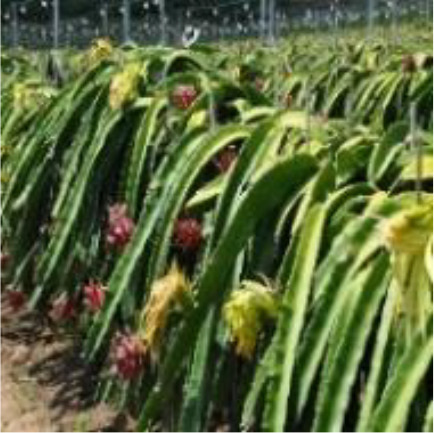	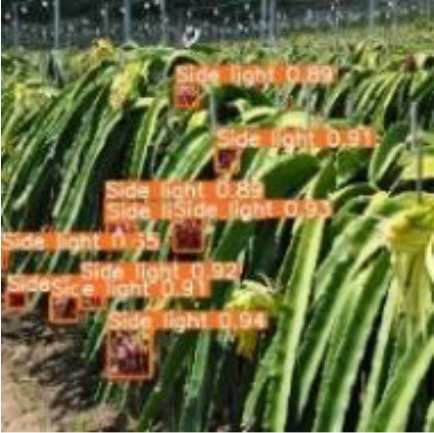	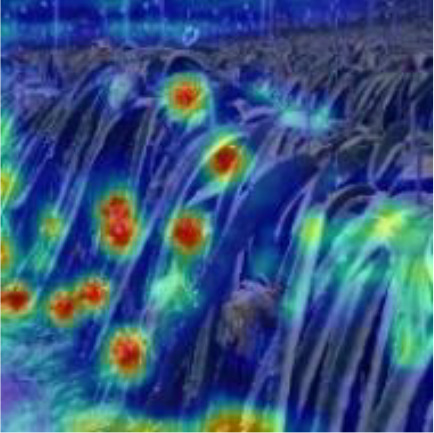	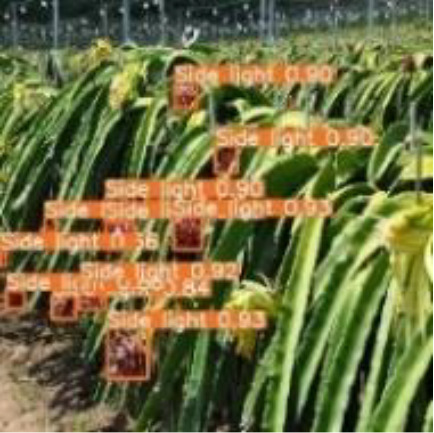	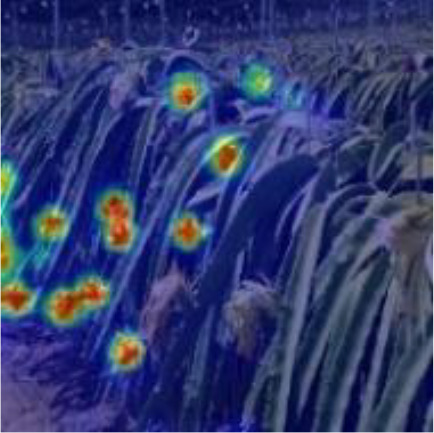

**Table 7:** Visual results of dragon fruit object detection on cloudy and night days.

Cloudy	The original image	Visual result of YOLOv5s		Visual result of Improved YOLOv5s	
Single fruit big object	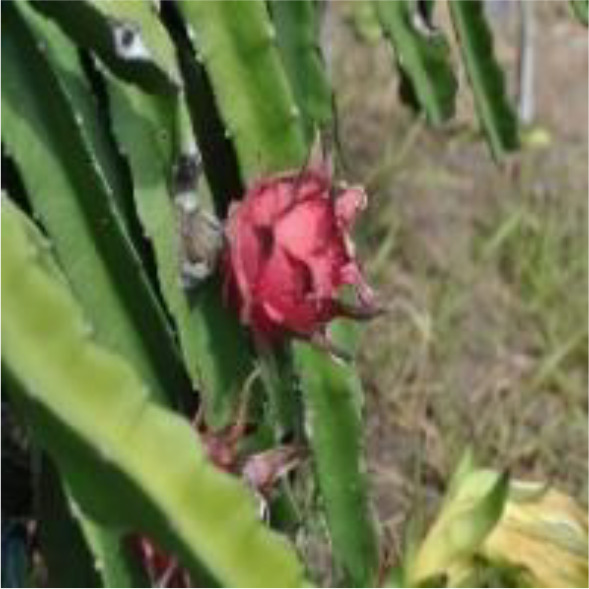	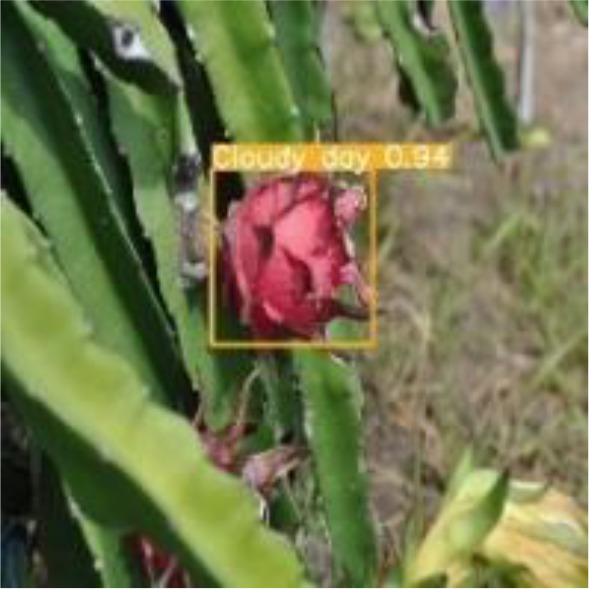	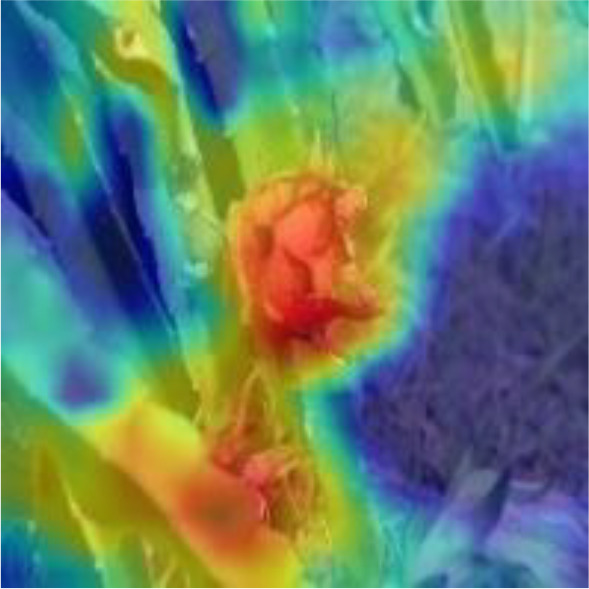	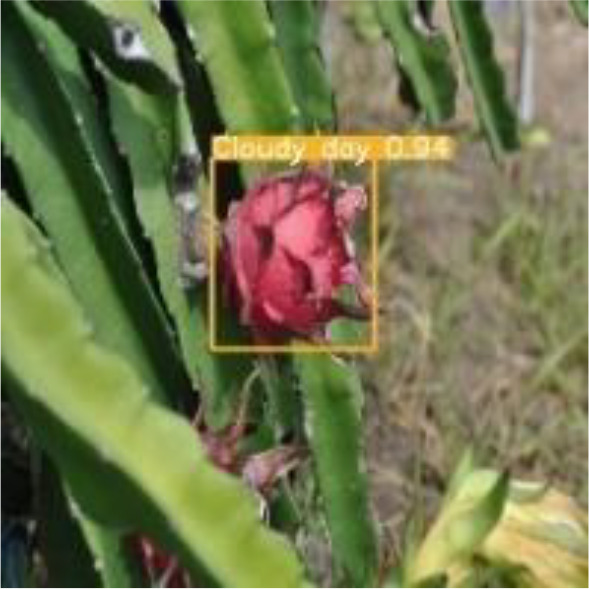	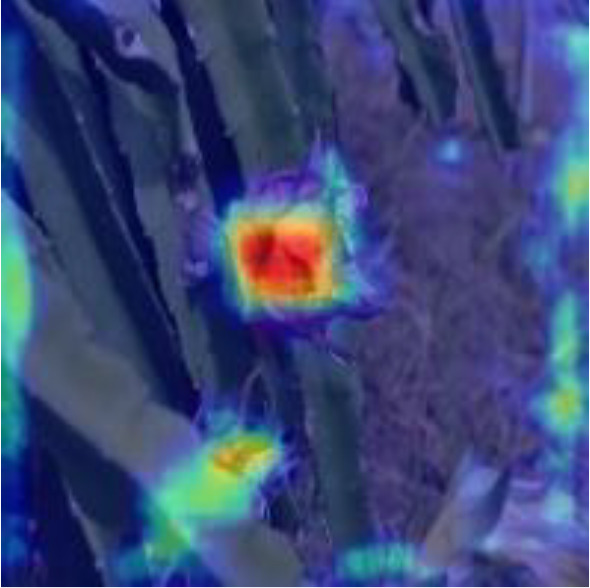
Many fruits in the goal	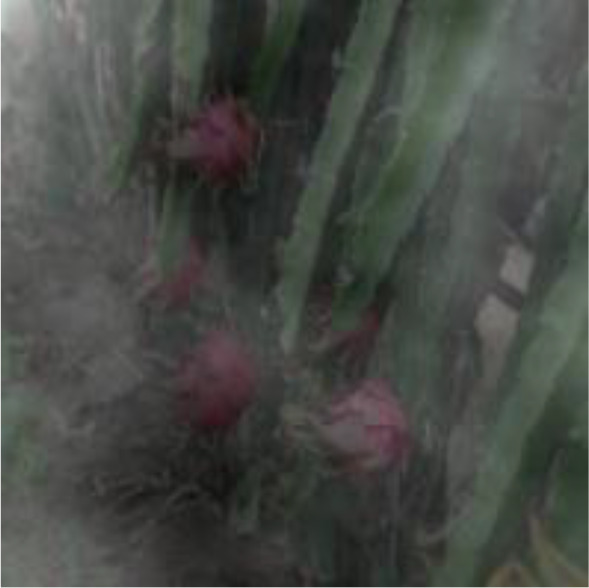	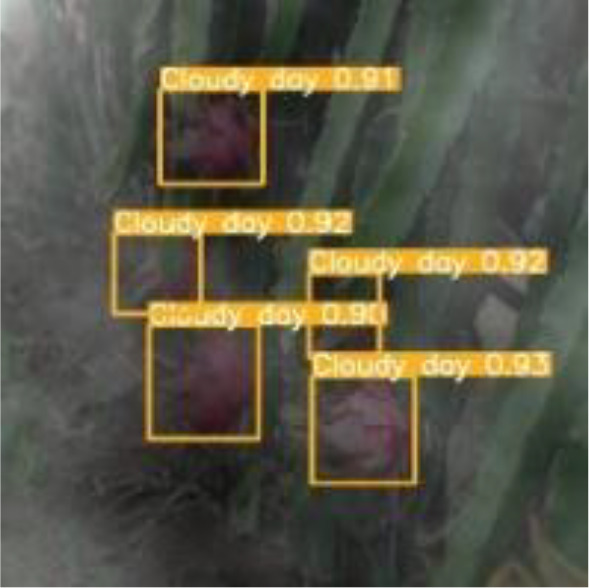	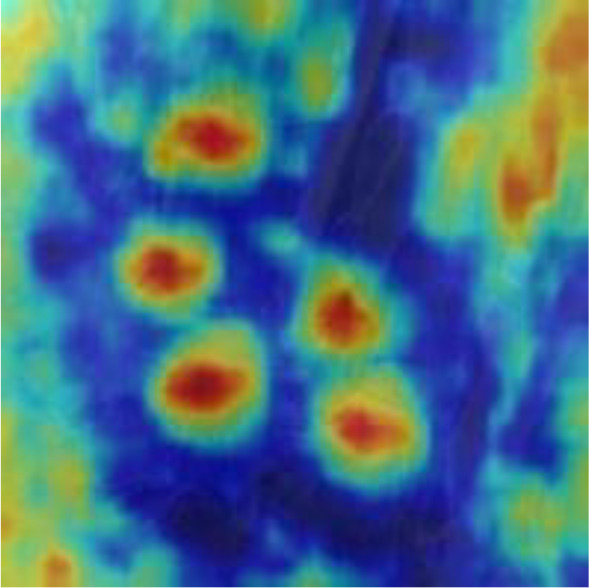	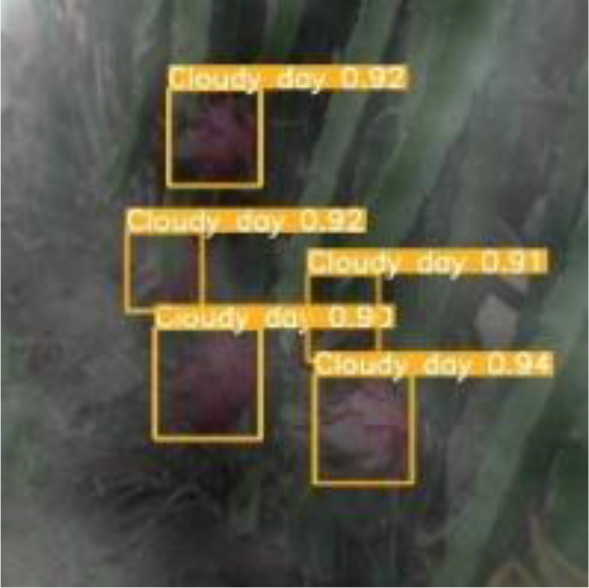	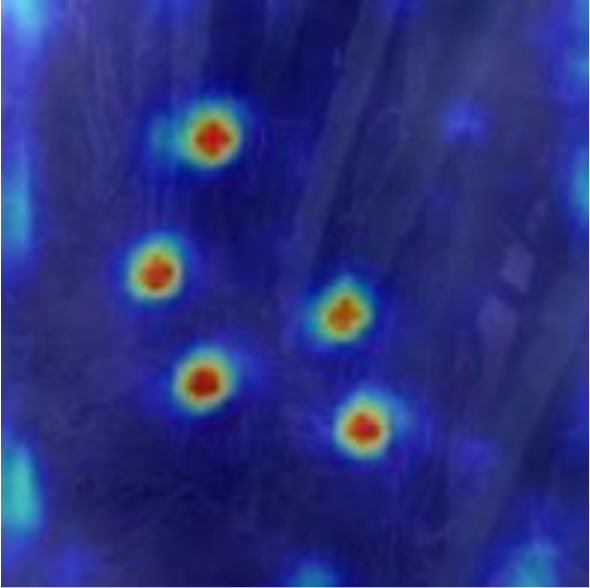
Dense small object	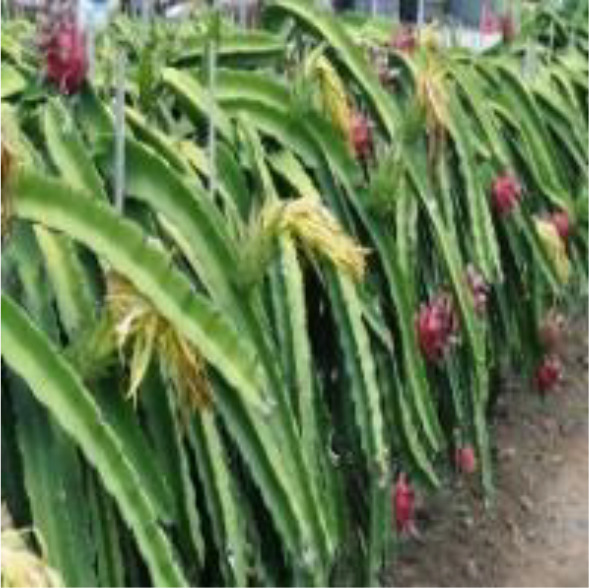	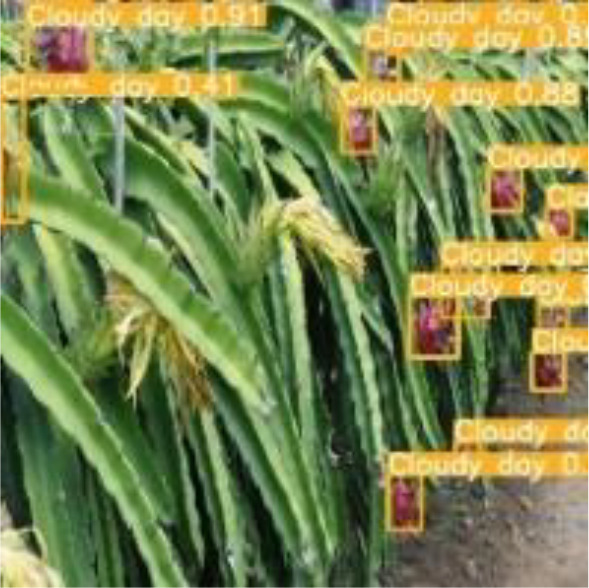	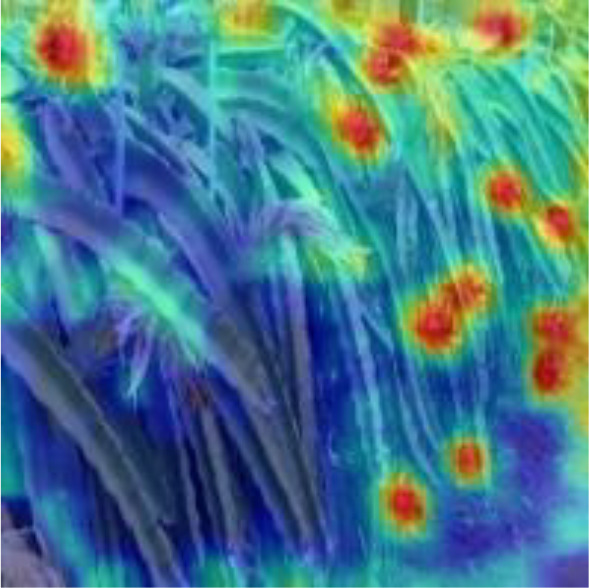	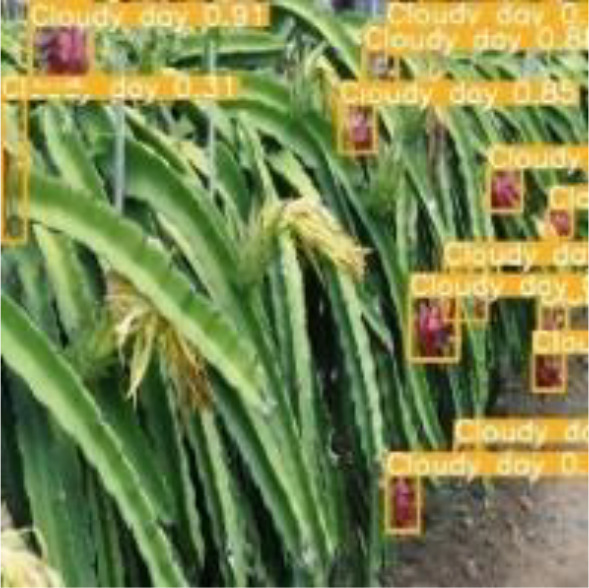	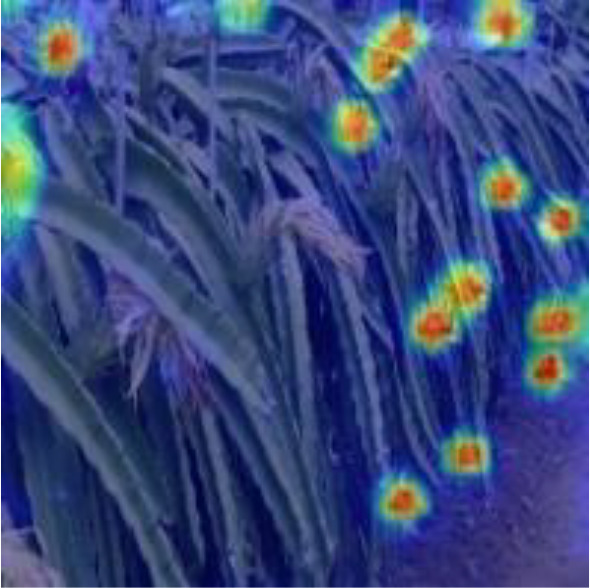
Night	The original image	Visual result of YOLOv5s		Visual result of Improved YOLOv5s	
Single fruit big object	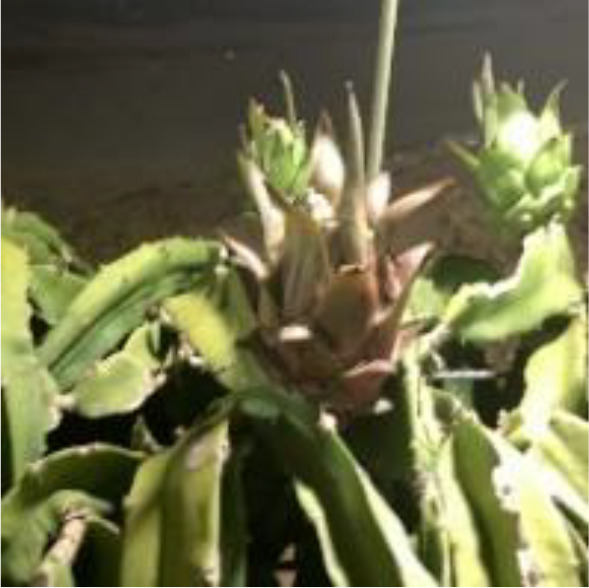	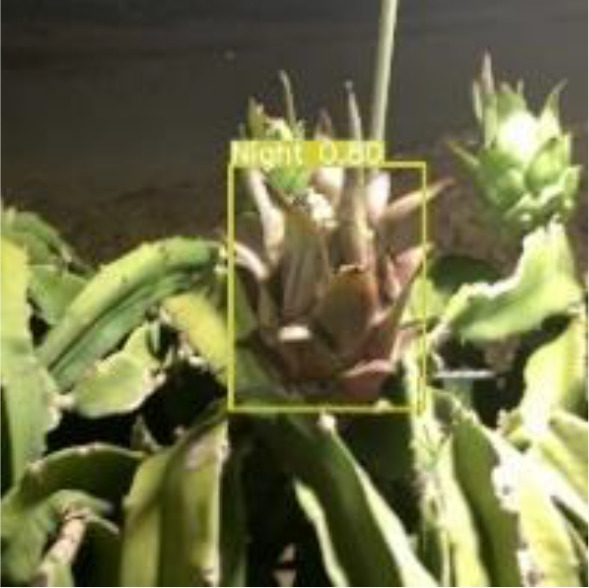	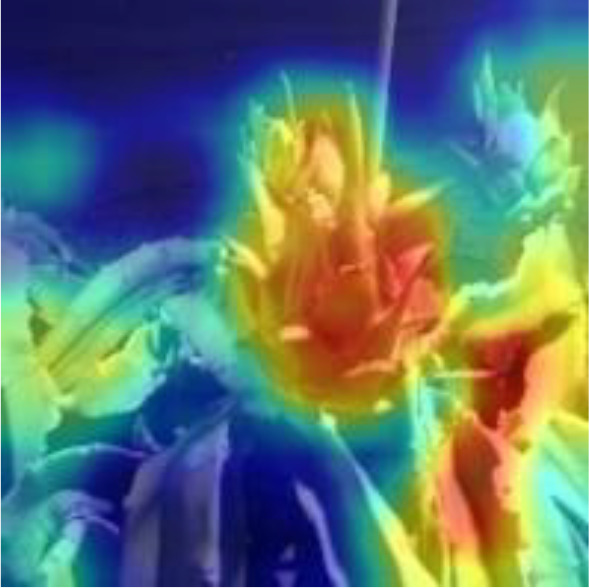	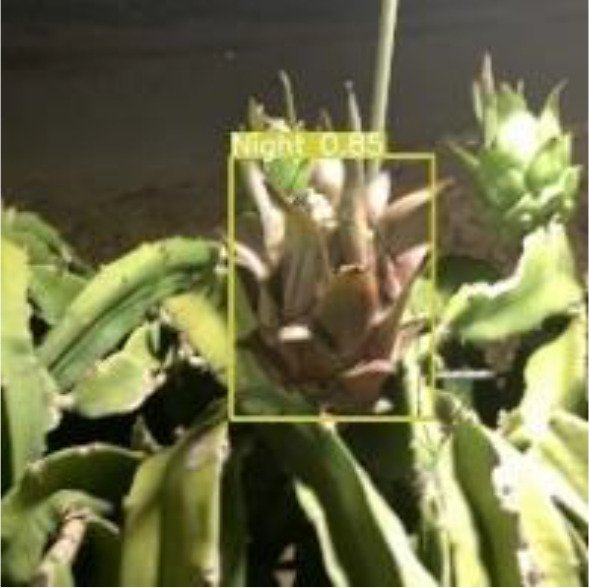	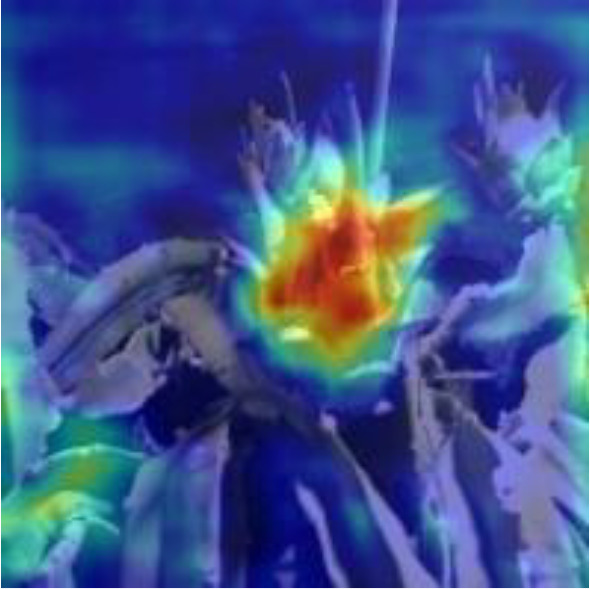
Many fruits in the goal	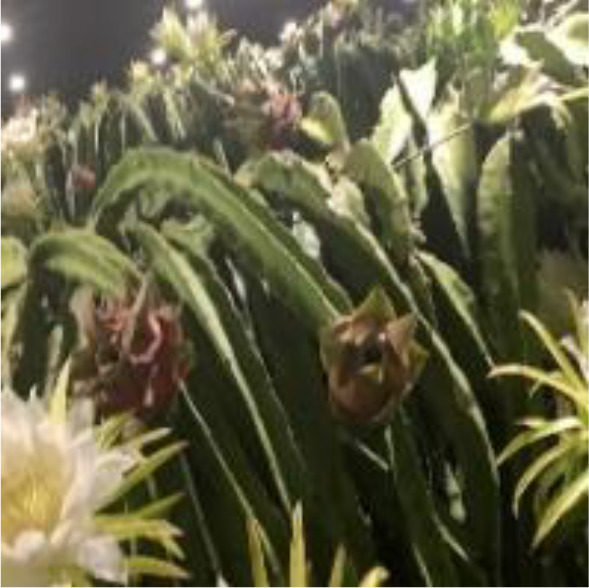	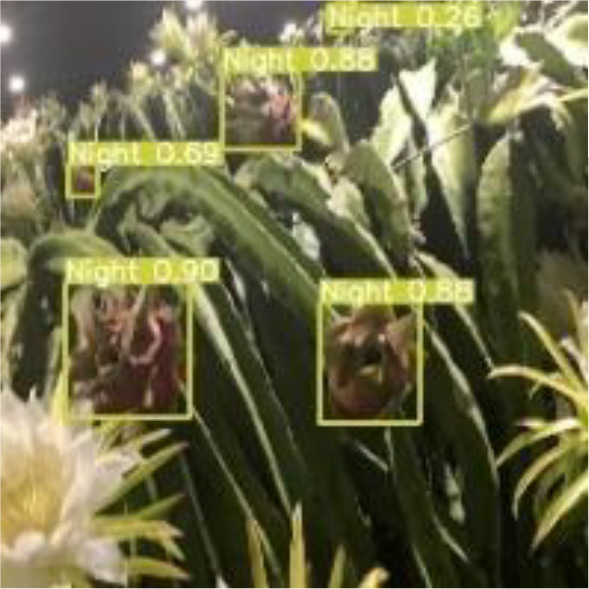	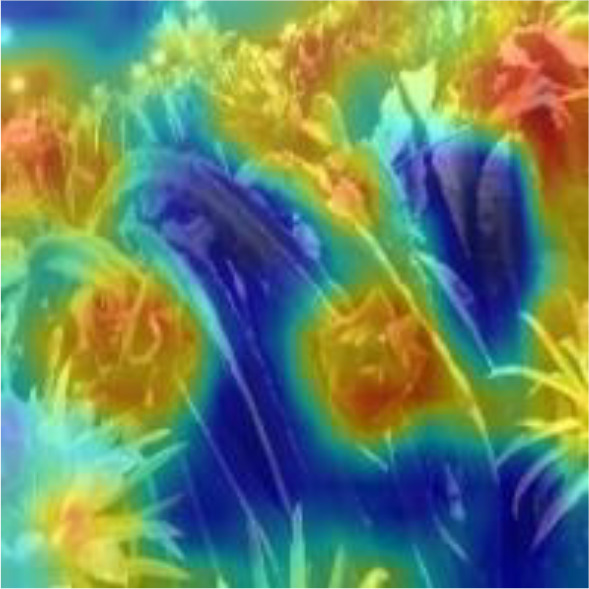	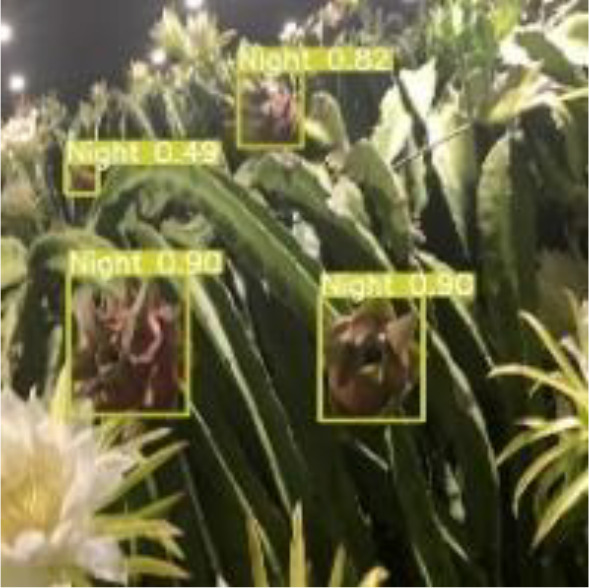	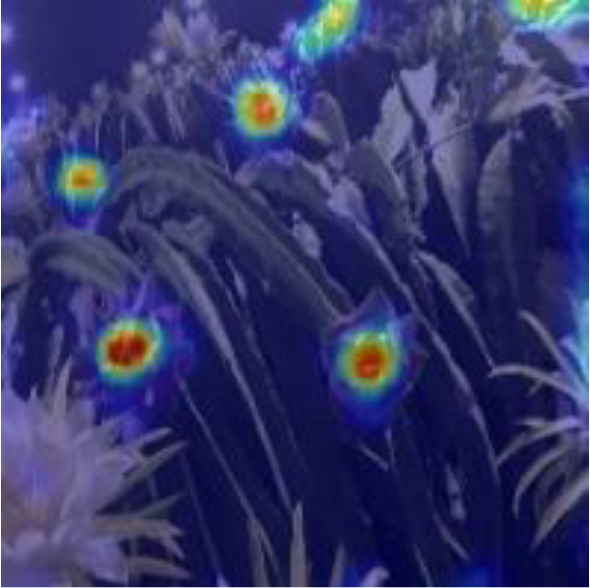
Dense small object	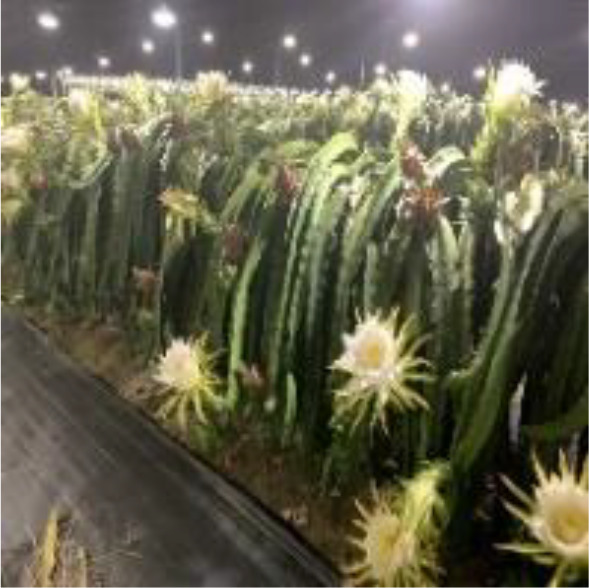	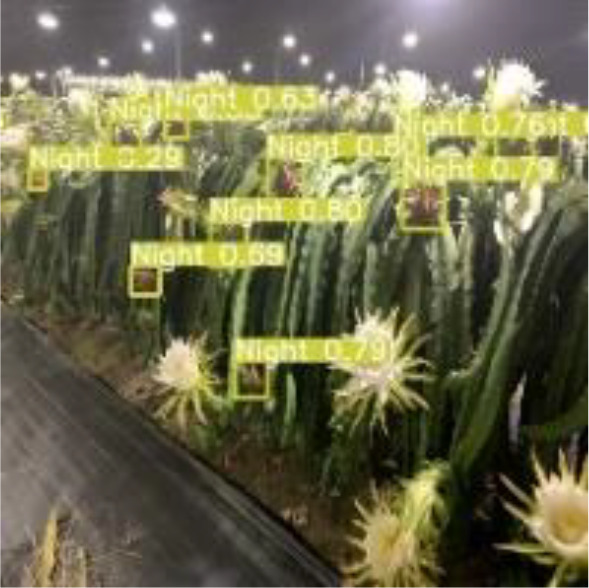	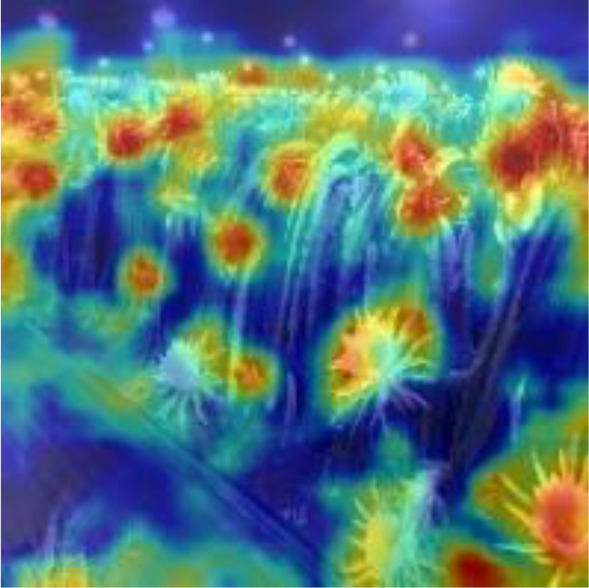	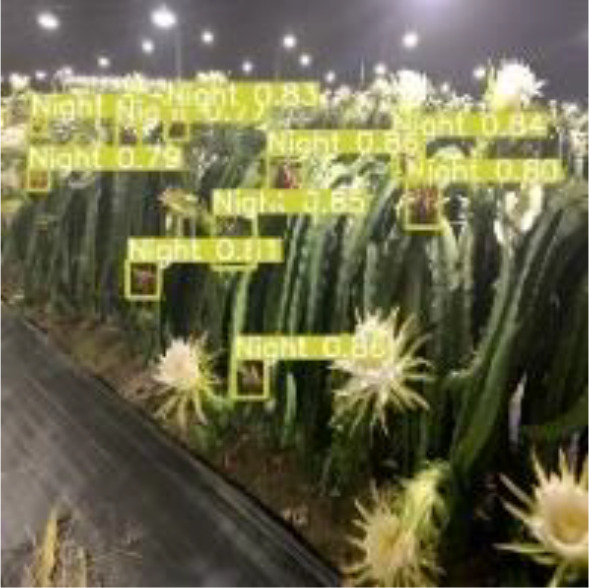	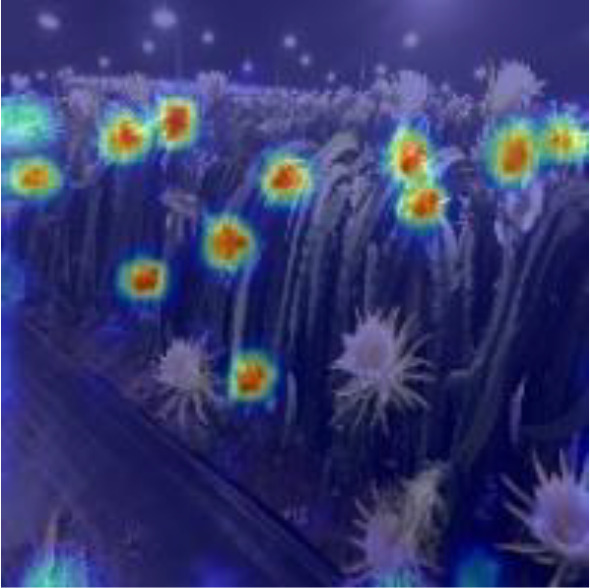

Video images of the dragon fruit orchard taken by UAV were used for detection to test further the real-time detection performance of the improved model on dragon fruit. UAV (model: Phantom 4 RTK) was used to shoot video images of the dragon fruit orchard at a low altitude of 1 meter in the daytime and night, with a resolution of 1280×720. The proposed improved model was used to detect the dragon fruits in video images. The results showed that the dragon fruits in the video images could be effectively detected. The image pre-processing time of single frame video is 0.6 ms, the reasoning time is 17.0 ms, and the post-processing time is 1.9 ms. The results further verify the strong robustness of the improved algorithm and provide technical support and research basis for deploying the algorithm on mobile devices and developing the vision system of orchard monitoring and picking equipment in the later stage.

## Conclusions

Aiming at the all-weather object detection of dense trellis planting of dragon fruit in a complex environment, a detection method that integrated a lightweight network and attention mechanism was proposed in this study. Firstly, dragon fruit data sets were constructed in complex natural environments. Second, the lightweight ghost module and CAM were integrated into the YOLOv5s network structure, while a bidirectional weighted feature pyramid network was constructed in the neck part of the network. Finally, the SIoU loss function was used to replace the loss function of the original network model to improve the convergence speed during model training.

The *mAP* value of the testing sets for dragon fruit detection by this method was 97.4%, *P* was 96.4%, *R* was 95.2%, model size was 11.5 MB, Params was 5.2 M, and FLOPs was 11.4 G. Compared with the original YOLOv5s network, the model size, Params and FLOPs of the model were reduced by 20.6%, 18.75% and 27.8%, respectively, and the *mAP* of the model improved by 1.1%. The improved model has a lighter structure and better detection performance. Using this model, the *AP* of dragon fruit was 99.5%, 97.3%, 98.5%, 95.5% and 96.1% under front light, backlight, side light, cloudy day and night, respectively. The detection performance could meet the requirements of all-weather detection of dragon fruit and had good robustness. The model was used to test video images with a resolution of 1280×720. The results showed that the pre-processing time of a single frame video image was 0.6 ms, the reasoning time was 17.0 ms, and the post-processing time was 1.9 ms. The model had good application potential in intelligent operations, such as orchard counting and yield measurement, fruit disease and insect pest monitoring by low-altitude UAV and precise picking in the field based on the picking robot.

The next research will mainly apply the existing model to practical tasks, such as orchard counting and yield measurement, fruit disease and insect pest monitoring by low-altitude UAV and precise picking in the field based on the picking robot. The data enhancement method and model detection performance will continue to be optimised to improve the detection accuracy of the model further.

## Data availability statement

The original contributions presented in the study are included in the article/supplementary material. Further inquiries can be directed to the corresponding authors.

## Author contributions

BZ designed and performed the experiment, selected the algorithm, analyzed the data, trained the algorithms, and wrote the manuscript. BZ, RW, CY, YX, and MF collected data. HZ monitored the data analysis. WF and HZ conceived the study and participated in its design. All authors contributed to this article and approved the submitted version.

## Funding

This work was supported by the Key R&D Projects in Hainan Province (Grant No. ZDYF2022XDNY231), the National Natural Science Foundation of China (Grant No. 32160424) and the Key R&D Projects in Hainan Province (Grant No. ZDYF2020042).

## Acknowledgments

The authors would like to thank their schools and colleges, as well as the funding of the project. All support and assistance are sincerely appreciated. Additionally, we sincerely appreciate the work of the editor and the reviewers of the present paper.

## Conflict of interest

The authors declare that the research was conducted in the absence of any commercial or financial relationships that could be construed as a potential conflict of interest.

## Publisher’s note

All claims expressed in this article are solely those of the authors and do not necessarily represent those of their affiliated organizations, or those of the publisher, the editors and the reviewers. Any product that may be evaluated in this article, or claim that may be made by its manufacturer, is not guaranteed or endorsed by the publisher.
